# Airway pressures generated by high flow nasal cannula in patients with acute hypoxemic respiratory failure: a computational study

**DOI:** 10.1186/s12931-025-03096-x

**Published:** 2025-01-08

**Authors:** Hossein Shamohammadi, Liam Weaver, Sina Saffaran, Roberto Tonelli, Marianna Laviola, John G. Laffey, Luigi Camporota, Timothy E. Scott, Jonathan G. Hardman, Enrico Clini, Declan G. Bates

**Affiliations:** 1 School of Engineering, University of Warwick, Coventry, CV4 7AL UK; 2 Respiratory Diseases Unit, Department of Medical and Surgical Sciences, University Hospital of Modena, University of Modena and Reggio Emilia, Modena, Italy; 3 Anaesthesia and Critical Care, Injury Inflammation and Recovery Sciences, School of Medicine, University of Nottingham, Nottingham, NG7 2UH UK; 4 Anaesthesia and Intensive Care Medicine, School of Medicine, Galway University Hospitals, University of Galway, Galway, H91 TK33 Ireland; 5 Intensive Care Medicine, Division of Asthma Allergy and Lung Biology, Guy’s and St Thomas’ NHS Foundation Trust, King’s College London, London, UK; 6 Centre for Biomechanics and Rehabilitation Technologies, Stoke on Trent, University of Staffordshire, Stoke-on-Trent, ST4 2DF UK; 7 Nottingham University Hospitals NHS Trust, Nottingham, NG7 2UH UK

**Keywords:** High flow nasal cannula, Non-invasive respiratory support, Mathematical modelling, Computer simulation, Positive end expiratory pressure

## Abstract

**Introduction and objectives:**

High flow nasal cannula (HFNC) therapy is an increasingly popular mode of non-invasive respiratory support for the treatment of patients with acute hypoxemic respiratory failure (AHRF). Previous experimental studies in healthy subjects have established that HFNC generates flow-dependent positive airway pressures, but no data is available on the levels of mean airway pressure (mP_aw_) or positive end-expiratory pressure (PEEP) generated by HFNC therapy in AHRF patients. We aimed to estimate the airway pressures generated by HFNC at different flow rates in patients with AHRF, whose functional lung volume may be significantly reduced compared to healthy subjects due to alveolar consolidation and/or collapse.

**Materials and methods:**

We developed a high-fidelity mechanistic computational model of the cardiopulmonary system during HFNC therapy using data from healthy subjects, and then measured the mP_aw_ and PEEP levels produced when different amounts of alveolar consolidation/collapse were incorporated into the model.

**Results:**

When calibrated to represent normal lung physiology in healthy subjects, our model recapitulates the airway pressures produced by HFNC at different flow rates in healthy volunteers who were breathing normally, with their mouths closed or open. When different amounts of alveolar consolidation/collapse are implemented in the model to reflect the pathophysiology of AHRF, the mP_aw_ and PEEP produced by HFNC at all flow rates increase as the functional lung volume decreases (up to a mP_aw_ of 11.53 and a PEEP of 11.41 cmH_2_O at 60 L/min with the mouth closed when 50% of the model’s alveolar compartments are non-aerated). When the model was matched to individual patient data from a cohort of 58 patients with AHRF receiving HFNC at 60 L/min, the mean (standard deviation) of the mP_aw_ / PEEP produced by HFNC in the models of these patients was 8.56 (1.50) / 8.92 (1.49) cmH_2_O with mouths closed, and 1.73 (0.31) / 1.36 (0.36) cmH_2_O with mouths open.

**Conclusions:**

Our results suggest that the airway pressures produced by HFNC in patients with AHRF could be higher than is currently assumed based on experimental data from healthy subjects, particularly in patients whose mouths remain closed. Higher levels of PEEP could be beneficial if they lead to alveolar recruitment and improved lung compliance, but could cause alveolar overdistension if they do not, motivating the close monitoring of the effects of HFNC on lung mechanics. Further clinical studies are warranted to directly measure the airway pressures produced by HFNC in patients with different severities of AHRF.

**Supplementary Information:**

The online version contains supplementary material available at 10.1186/s12931-025-03096-x.

## Introduction

High flow nasal cannula (HFNC) therapy delivers heated and humidified air or oxygen to patients at high flow rates, typically between 20 and 60 L/min, via a nasal cannula interface [1, 2]. HFNC is an increasingly popular form of non-invasive respiratory support for the treatment of acute respiratory failure, particularly since the COVID-19 pandemic [3–6]. In contrast to other forms of non-invasive respiratory support such as continuous positive airway pressure (CPAP) or non-invasive ventilation (NIV), which use a tightly sealed mask or helmet to deliver positive pressure to the lungs, HFNC therapy provides a set flow rate of air or oxygen via a nasal cannula interface, with either symmetrical [1] or asymmetrical prongs [7, 8].

The ability of HFNC therapy to generate a flow-rate-dependent positive airway pressure is well accepted among clinicians. However, estimates made to date of the level of airway pressures produced by HFNC at different flow rates are based exclusively on measurements made in subjects who were not suffering from acute respiratory failure. In healthy subjects whose mouths were closed, a HFNC flow rate of 60 L/min (typically the maximum used in clinical practice) produced average mean airway pressures of 6.12 cmH_2_O in [9] and 6.78 cmH_2_O in [10], and an average expiratory pressure of 7.4 cmH_2_O in [11]. Increased mean airway pressure compared to spontaneous breathing has been measured in patients with chronic obstructive pulmonary disease (COPD) at various HFNC flow rates up to 50 L/min. At 50 L/min, mean airway pressure increased to 3.01 ± 1.03 cmH_2_O [12]. Similarly, in another study involving hypercapnic patients with interstitial lung disease (ILD), a flow-dependent increase in mean airway pressure was measured in the nasopharyngeal space, reaching a maximum of 3.1 ± 1.5 cmH_2_O at 50 L/min [13].- See Table S1 of the Supplementary Material for a full review of relevant data from previous studies.

In contrast to the situations considered above, the functional lung volume is reduced in acute respiratory failure. Gattinoni [14] introduced the concept of the “baby lung” in acute respiratory distress syndrome (ARDS), based on computed tomography examinations which showed that some ARDS patients could have normally aerated lung tissue corresponding to the dimensions of the lung of a 5- to 6-year-old child (300–500 g of aerated tissue). Based on the standard relationship between pressure and volume in ideal gases (Pressure $\:\times\:$ Volume = Mass of gas $\:\times\:$ Gas constant $\:\times\:$ Temperature), we hypothesised that if the flow of air delivered by HFNC into the lungs is similar, smaller functional lung volumes in patients with AHRF could lead to HFNC providing higher airway pressures than that expected based on data from healthy subjects.

## Methods

### Patient and public involvement

No patients were involved in the study, all results are based on modelling of data from previously published studies.

### Modelling a cohort of healthy subjects receiving HFNC

To investigate the relationship between functional lung volume and airway pressures produced by HFNC, we adapted a multi-compartmental computational simulator, previously employed to simulate patients with different conditions, such as COVID-19 [15], chronic obstructive pulmonary disease (COPD) [16], ARDS [17, 18] and AHRF [19]. The simulator offers several advantages including the ability to define multiple alveolar compartments with individually configurable mechanical characteristics such as alveolar collapse, consolidation and stiffening, gas-exchange disruption, pulmonary vasoconstriction, vasodilation, and airway obstruction. This allows for the representation of various clinical features including acute lung injury, ventilation-perfusion mismatch, physiological shunt, alveolar gas trapping with intrinsic positive end-expiratory pressure (iPEEP), and reopening of collapsed alveoli [20, 21]. Key mechanisms involved in the application of HFNC therapy, including carbon dioxide clearance from dead space, gas leakage, a friction factor for turbulent flow, and increases in airway resistance at high flow rates are included in the model presented here – full details are provided in Sect. 4 of the Supplementary Material.

We initially adapted this computational simulator to create a virtual cohort of ten subjects whose characteristics were representative of participants in four previous studies reporting mean airway pressures in healthy subjects at multiple HFNC flow rates [9, 10, 22, 23]. This virtual cohort consists of an equal number of males and females, with an average age of 33 years, height of 170 cm, and weight of 74 kg, all falling within the normal BMI range. Detailed information regarding the cohort is presented in Table S2 in the supplementary material. Healthy lung physiology and respiratory effort are simulated in all cases, with compliance and airway resistance values varying within normal ranges.

### Modelling reductions in functional lung volume in patients receiving HFNC therapy

To simulate alveolar consolidation/collapse (i.e., compartments with no participation in ventilation), the inlet resistance of a specified number of the alveolar compartments in the model is increased to a large value to preclude any airflow to the alveolus. In this part of the investigation, all other parameters pertaining to the subjects including FiO_2_ were left unaltered, ensuring that the results are focused exclusively on elucidating the influence of the size of the functional lung volume on the pressures generated by HFNC.

### Modelling a cohort of AHRF patients receiving HFNC therapy

To estimate the PEEP produced by HFNC in actual AHRF patients, data were extracted from two studies [24, 25] reporting data on 60 non-COVID-19 patients with moderate-to-severe AHRF, conducted within a respiratory intensive care unit at the University Hospital of Modena, Italy, from 2016 to 2021. These comprised a comprehensive set of physiological measurements for each patient during their HFNC therapy, including gender, age, height, weight, fraction of inspired oxygen, and HFNC flow rate, all of which served as inputs for the cardiopulmonary simulator. Using global optimization algorithms, the simulator’s parameters were calibrated so that its outputs matched as closely as possible the responses of individual patients to HFNC therapy, encompassing partial pressures of oxygen (PaO_2_) and carbon dioxide (PaCO_2_) in the arterial blood, oesophageal pressure swings (ΔP_es_, measured by a dedicated oesophageal pressure transducer), and expiratory tidal volume (VT, measured by numerical integration of respiratory flow measured by a pneumotachograph) – for full details of the model matching process, see the supplementary material. Two patients were excluded from the analysis due to concerns regarding abnormally high VT measurements, potentially stemming from erroneous integration of a portion of the flow directed to the patient into the integrated flow signal.

### Calculating PEEP and mean airway pressure during HFNC therapy

Previous experimental studies [9, 10, 22, 23], have measured mean airway pressure (mP_aw_) produced by HFNC at the participants’ pharynx. In our model, tracheal pressure serves as the reference for calculating both PEEP and mP_aw_. Specifically, PEEP is determined as the positive end-expiratory pressure calculated at the trachea, while mP_aw_ represents the mean tracheal pressure throughout one complete breathing cycle.

## Results

### The cardiopulmonary simulator recapitulates airway pressures measured in healthy subjects receiving HFNC at different flow rates

The cardiopulmonary simulator effectively replicates airway pressures observed in healthy subjects undergoing HFNC therapy across various flow rates, in both mouth closed and open conditions. As shown in Table 1, the simulated mP_aw_ values closely match those reported in the experimental studies. Study [9] reported mP_aw_ in a closed-mouth condition at set flow rates ranging from 30 to 100 L/min, while another study [10] reported mP_aw_ in an open-mouth condition at set flow rates of 20, 40, and 60 L/min.

**Table 1:** Simulated Mean Airway pressure (mP_aw_) in 10 healthy subjects at different HFNC flow rates compared to data from [9, 10] (*S.D. not reported)

Mouth condition	HFNC flow rate (L/min)	Experimental mP_aw_±SD (cmH_2_O)	Simulated mP_aw_±SD (cmH_2_O)
**closed**	30	2.71 ± 0.74	2.75 ± 0.59
	40	3.79 ± 0.84	3.92 ± 0.80
	50	4.95 ± 1.09	5.03 ± 0.96
	60	6.12 ± 1.37	6.37 ± 1.12
	70	7.69 ± 1.46	7.80 ± 1.34
	80	9.00 ± 1.89	9.33 ± 1.74
	90	10.05 ± 2.11	10.62 ± 1.99
	100	11.88 ± 2.73	12.50 ± 2.63
**open**	20	0.18*	0.19 ± 0.06
	40	0.54*	0.57 ± 0.12
	60	0.75*	0.77 ± 0.16

### At fixed HFNC flow rates, PEEP increases as the functional lung volume decreases

In our model, at fixed HFNC flow rates, PEEP increases as the percentage of consolidated/collapsed lung volume increases (Table 2). For instance, at a HFNC flow rate of 60 L/min PEEP ranges from 6.19 ± 1.13 and 0.75 ± 0.19 cmH_2_O in healthy subjects to 11.41 ± 2.56 and 1.55 ± 0.51 cmH_2_O in subjects with 50% consolidated/collapsed lung, with the mouth closed and open, respectively.

**Table 2:** mP_aw_ and PEEP at different HFNC flow rates with varying percentages of consolidated/collapsed lung volume

HFNC flow rate (L/min)	Mouth condition	Pressures (cmH_2_O)	Lung consolidation/collapse (%)					
			0	10	20	30	40	50
**30**	**closed**	**mP**_**aw**_ **±SD**	2.75 ± 0.59	2.99 ± 0.65	3.28 ± 0.73	3.66 ± 0.84	4.18 ± 0.98	4.91 ± 1.18
		**PEEP ± SD**	2.62 ± 0.60	2.87 ± 0.66	3.17 ± 0.74	3.57 ± 0.84	4.10 ± 0.99	4.85 ± 1.18
**40**	**closed**	**mP**_**aw**_ **±SD**	3.92 ± 0.80	4.22 ± 0.88	4.62 ± 0.99	5.13 ± 1.14	5.84 ± 1.33	6.82 ± 1.59
		**PEEP ± SD**	3.72 ± 0.81	4.06 ± 0.89	4.47 ± 1.00	5.01 ± 1.14	5.73 ± 1.33	9.74 ± 1.60
	**open**	**mP**_**aw**_ **±SD**	0.57 ± 0.12	0.65 ± 0.15	0.72 ± 0.17	0.81 ± 0.20	0.93 ± 0.23	1.10 ± 0.28
		**PEEP ± SD**	0.54 ± 0.13	0.63 ± 0.17	0.71 ± 0.21	0.80 ± 0.19	0.92 ± 0.22	1.24 ± 0.46
**50**	**closed**	**mP**_**aw**_ **±SD**	5.03 ± 0.96	5.69 ± 1.14	6.20 ± 1.28	6.87 ± 1.46	7.78 ± 1.71	9.05 ± 2.06
		**PEEP ± SD**	4.87 ± 0.95	5.49 ± 1.15	6.03 ± 1.29	6.72 ± 1.47	7.65 ± 1.72	8.95 ± 2.06
**60**	**closed**	**mP**_**aw**_ **±SD**	6.37 ± 1.12	7.37 ± 1.41	8.01 ± 1.59	8.84 ± 1.81	9.96 ± 2.12	11.53 ± 2.55
		**PEEP ± SD**	6.19 ± 1.13	7.13 ± 1.42	7.80 ± 1.60	8.65 ± 1.83	9.81 ± 2.13	11.41 ± 2.56
	**open**	**mP**_**aw**_ **±SD**	0.77 ± 0.17	0.86 ± 0.20	0.95 ± 0.23	1.08 ± 0.26	1.25 ± 0.31	1.47 ± 0.37
		**PEEP ± SD**	0.75 ± 0.19	0.83 ± 0.22	0.93 ± 0.23	1.08 ± 0.28	1.24 ± 0.31	1.55 ± 0.51

### PEEP in models of 58 AHRF patients receiving HFNC at 60 L/min

**Fig. 1:**
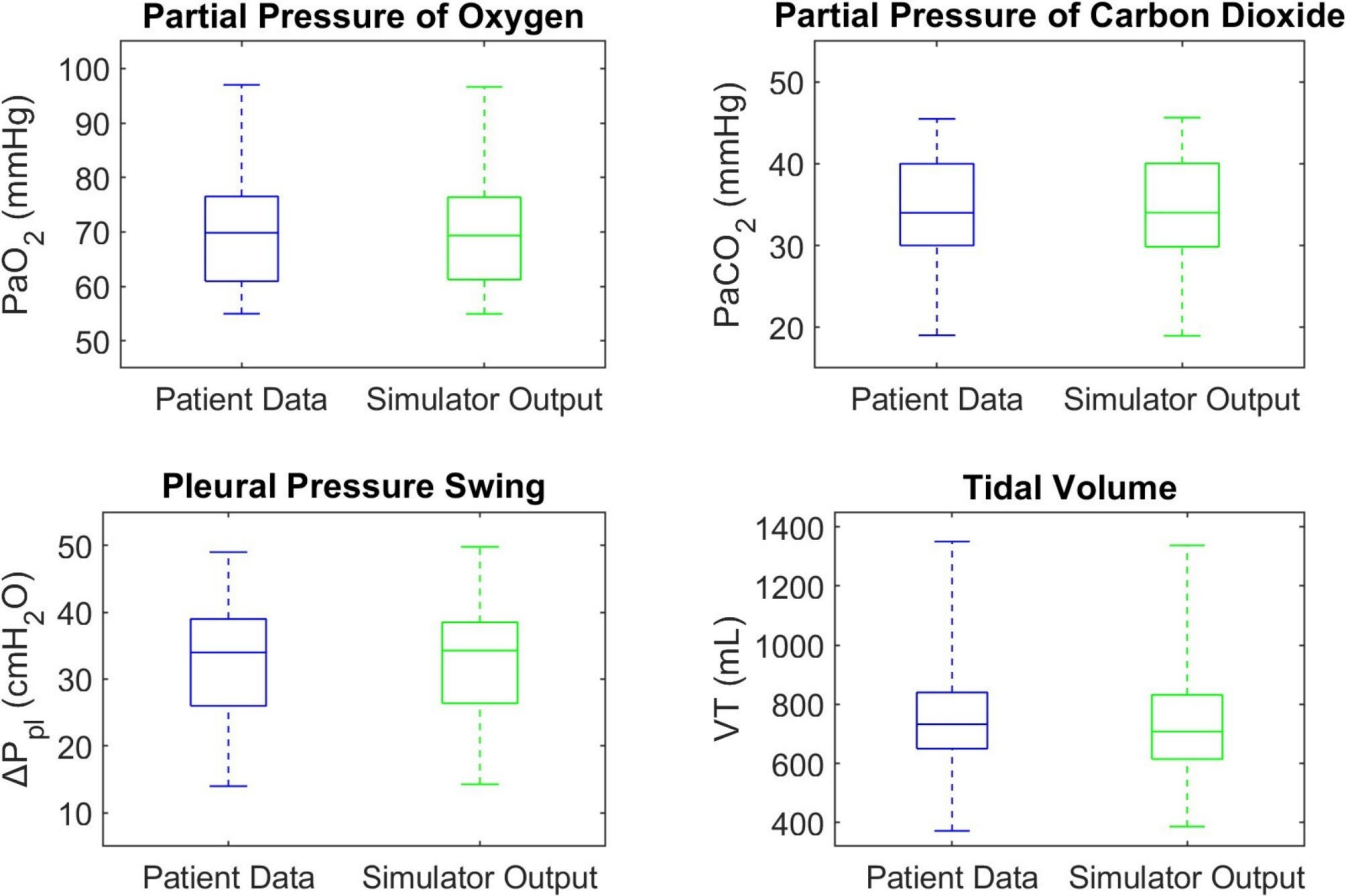
Patient data distributions compared to simulator outputs while on HFNC, median, interquartile ranges, and actual ranges

Figure 1 compares the simulator outputs against individual measurements of PaO_2_, PaCO_2_, tidal change in pleural pressure (ΔP_pl_), and VT in 58 patients during HFNC therapy at 60 L/min flow rate [24, 25]. In the case of ΔP_pl_, we compare the actual tidal change in pleural pressure obtained from the model with its surrogate, ΔP_es_. Across the cohort, the mean absolute percentage errors between the patient data and simulator outputs were 0.5% for PaO_2_, 0.6% for PaCO_2_, 2.5% for ΔP_pl_, and 4.5% for VT.

**Fig. 2:**
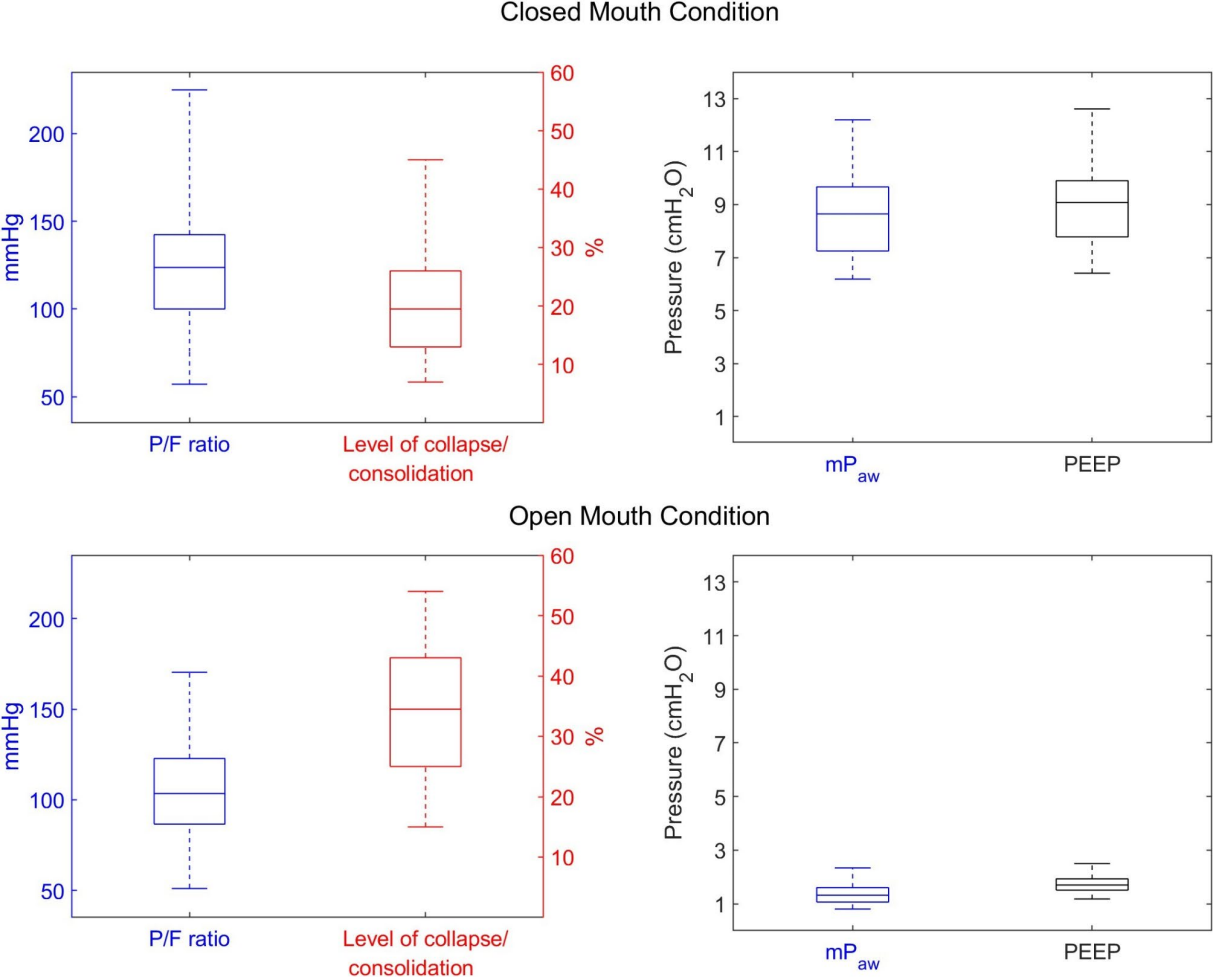
P/F ratio, level of consolidated/collapsed lung, mP_aw_, and PEEP in the 58 models of patients receiving HFNC at 60 L/min under closed and open mouth condition, median, interquartile ranges, and actual ranges

The mean (standard deviation) of mP_aw_ and PEEP calculated in the models across the cohort of 58 patients with AHRF, all of whom were receiving HFNC therapy at 60 L/min with their mouths closed, was 8.56 (1.50) and 8.92 (1.49) cmH_2_O, respectively. When simulated in the open mouth condition, the mP_aw_ and PEEP recorded in the model were 1.73 (0.31) and 1.36 (0.36) cmH_2_O, respectively. The mean (standard deviation) percentage of consolidated/collapsed compartments in the models was 20 (8.53) in the closed mouth condition and 34.5 (10.9) in open mouth condition (see Fig. 2).

## Discussion

The ability of HFNC to produce a flow-dependent positive airway pressure in healthy subjects has been conclusively demonstrated in multiple studies [9, 10, 22, 23]. However, no studies to date have measured the PEEP produced by HFNC in actual AHRF patients. The results presented here suggest that the PEEP produced by HFNC in AHRF patients may be 40% higher than that produced in healthy subjects, on average, with significantly greater differences being possible in some patients. These results were produced using a model which, when calibrated to represent healthy lung physiology, reproduces the pressures measured in previous human studies at multiple HFNC flow rates, in both mouth closed and open conditions. When this model is adjusted to reflect different levels of alveolar consolidation/collapse within the lung, PEEP increases with the amount of non-functional lung, at all flow rates. When the model parameters are adjusted so that they match data from 58 individual moderate-to-severe AHRF patients receiving HFNC therapy at 60 L/min [24, 25], it suggests the average PEEP was 8.92 cmH_2_O across this cohort, with some patients receiving a PEEP of more than 12 cmH_2_O.

Interestingly, a recent experimental study of the effect of different prong/nare ratios in HFNC which used a three-dimensionally printed bench model measured a PEEP of approximately 11 cmH_2_O at 60 L/min flow rate and a prong/nare ratio of 0.51 [26]. Greater occlusion of the nares by the nasal cannula prongs can increase the resistance to flow and lead to higher generated pressure – in the studies involving the 58 AHRF patients whose data is used here, the largest prongs available were used in an attempt to provide the maximum possible level of support (all patients subsequently failed HFNC therapy and were consecutively given a trial of NIV. Another recent bench study using a mechanical lung respiratory simulation system with a prong/nare ratio of 0.63 reported a PEEP value of 10.3 cmH_2_O at a HFNC flow rate of 60 L/min [27], while PEEP values of up to 36 cmH_2_O were measured at flows of 60 L/min in a mechanical model of the paediatric lung [28].

Two recent physiological studies comparing the effects of HFNC at 50–60 L/min flow rates with NIV or CPAP [29, 30] in AHRF patients found large differences in terms of gas exchange and end-expiratory lung volume (EELV). PEEP was not measured during HFNC in these studies, but the PEEP applied during NIV (10–12 cmH_2_O plus additional inspiratory pressure support) and CPAP (14 cmH_2_O) were higher than our estimates for that produced by HFNC. These differences, together with the fact that some patients receiving HFNC may have had their mouths open (data not reported), may explain the differences in gas exchange and EELV found in those studies. Lower PEEP levels were measured previously in healthy volunteers whose mouths were open during HFNC (Table S1) – our results suggest that the reduction in PEEP caused by opening the mouth may be even greater in patients with AHRF.

In our models of patients with AHRF, HFNC produces higher pressures than previously observed in healthy subjects because the functional volume of the lungs is reduced due to alveolar consolidation/collapse – across the models of 58 AHRF patients the average level of non-aerated lung volume produced by the process of matching the models to data of patients was 20%. In the context of invasive mechanical ventilation of patients with ARDS, it is by now well established that ventilator-induced lung injury (VILI) essentially corresponds to the application of excessive stress and strain to the “baby lung” [14] – the smaller the “baby lung” the greater the potential for VILI. Appropriate levels of PEEP may keep open portions of the lung that are prone to consolidation/collapse, increasing the resting end expiratory lung volume and reducing stress and strain [31]. If PEEP does not recruit collapsed alveoli, however, it may overdistend already open alveoli, increasing stress and strain [32]. In patients with AHRF who are spontaneously breathing, the PEEP effect of HFNC primarily arises from increased respiratory system resistance during the expiratory phase. This prevents the collapse of alveoli during exhalation and leads to an improvement in overall end-expiratory lung impedance with reduced gas shifting and pendelluft [33]. However, it can also potentially cause overdistension, particularly in non-dependent lung regions, when the lung is non-homogeneous. In this context, the detrimental effects of excessive PEEP are likely to be more dependent on the type of lung injury, and to be primarily generated during the expiratory phase when elastic recoil increases airway resistance. Our study suggests that managing this difficult balancing act may be an important element of achieving positive outcomes in the context of spontaneously breathing patients receiving non-invasive respiratory support therapies such as HFNC.

Our study has some limitations. The model was calibrated to patient data from two studies conducted at the same centre, involving patients with moderate-to-severe AHRF, all of whom were subsequently escalated to non-invasive or invasive mechanical ventilation. No data were available with which to directly estimate the level of alveolar consolidation/collapse in these patients – the size of the functional lung volume used in our models is the result of adjusting its parameters so that it optimally reproduces the measurements of PaO_2_, PaCO_2_, oesophageal pressure swing and tidal volume made in each patient.

## Conclusions

The results of this study suggest that the positive airway pressures produced by HFNC in patients with AHRF who have reduced functional lung volumes due to alveolar consolidation/collapse might be significantly higher than is currently assumed based on the available experimental data from healthy subjects. In our model, at fixed HFNC flow rates, PEEP increased as the functional lung volume decreased. Higher levels of PEEP could be beneficial if they lead to alveolar recruitment and improved lung compliance, but if they do not produce recruitment then they could increase the risk of lung injury due to alveolar overdistension. While our results are logical consequences of standard gas pressure-volume relationships, further confirmatory clinical studies are warranted to directly measure the airway pressures produced by HFNC in patients with AHRF.

## Electronic supplementary material

Below is the link to the electronic supplementary material.

Supplementary Material 1

## Data Availability

All data generated or analysed during this study are included in this published article and its supplementary information files.

## Abbreviations

HFNC: High Flow Nasal Cannula
AHRF: Acute Hypoxemic Respiratory Failure
mP_aw_: Mean Airway Pressure
PEEP: Positive End Expiratory Pressure
CPAP: Continuous Positive Airway Pressure
COPD: Chronic Obstructive Pulmonary Disease
ARDS: Acute Respiratory Distress Syndrome
ICU: Intensive Care Unit
PaO_2_: Partial Pressure of Arterial Oxygen
PaCO_2_: Partial Pressure of Arterial Carbon Dioxide
ΔPpl: Tidal Change in Pleural Pressure
VT: Tidal Volume
NIV: Non-Invasive Ventilation
VILI: Ventilator Induced Lung Injury
EELV: End Expiratory Lung Volume
