# Supplementary material for: Airway pressures generated by high flow nasal cannula in patients with acute hypoxemic respiratory failure: a computational study
Source: Respir Res. 2025 Jan 8;26:9. doi: 10.1186/s12931-025-03096-x (PMC11715915; doi:10.1186/s12931-025-03096-x)
Supplement: Supplementary file 1 — Supplementary Material 1 [file 12931_2025_3096_MOESM1_ESM.docx]

**Airway pressures generated by high flow nasal cannula in patients with acute hypoxemic respiratory failure: A computational study**

Hossein Shamohammadi^1^, Liam Weaver^1^, Sina Saffaran^1^, Roberto Tonelli^2^, Marianna Laviola^3^, John G. Laffey^4^, Luigi Camporota^5^, Timothy E. Scott^6^, Jonathan G. Hardman^3,7^, Enrico Clini^2^*, and Declan G. Bates^1^

Affiliations:

1. School of Engineering, University of Warwick, Coventry CV4 7AL, UK.

2. Respiratory Diseases Unit, Department of Medical and Surgical Sciences, University Hospital of Modena, University of Modena and Reggio Emilia, Modena, Italy.

3. Anaesthesia and Critical Care, Injury Inflammation and Recovery Sciences, School of Medicine, University of Nottingham, Nottingham NG7 2UH, UK.

4. Anaesthesia and Intensive Care Medicine, Galway University Hospitals and School of Medicine, University of Galway, H91 TK33, Ireland.

5. Intensive Care Medicine, Guy’s and St Thomas’ NHS Foundation Trust and Division of Asthma Allergy and Lung Biology, King’s College London, UK.

6. Academic Department of Military Anaesthesia and Critical Care, Royal Centre for Defence Medicine, ICT Centre, Birmingham, B15 2SQ, UK.

7. Nottingham University Hospitals NHS Trust, Nottingham NG7 2UH, UK.

***Corresponding author:** enrico.clini@unimore.it (+39 059 4222918)

**Supplementary Material**

Table of Contents

[1- ICSM Pulmonary Model 1](#_Toc169178069)

[2- Modelling Spontaneous Breathing 10](#_Toc169178070)

[3- Calculating Lung Mechanics Parameters 11](#_Toc169178071)

[3.1 Compliance 11](#_Toc169178072)

[3.2 Transpulmonary Pressure 11](#_Toc169178073)

[3.3 Pleural Pressure 12](#_Toc169178074)

[3.4 Baseline End Expiratory Lung Volume 12](#_Toc169178075)

[3.5 Total Lung Strain 12](#_Toc169178076)

[3.6 Total Lung Stress 12](#_Toc169178077)

[3.7 Mechanical Power 12](#_Toc169178078)

[3.8 Driving Pressure 13](#_Toc169178079)

[4- Modelling High Flow Nasal Cannula Therapy 14](#_Toc169178080)

[4.1 Experimental studies measured airway pressures 14](#_Toc169178081)

[4.2 Creating a Virtual Cohort of Healthy Subjects 16](#_Toc169178082)

[4.3 Estimating leakage by calibrating the model to experimental data 17](#_Toc169178083)

[4.4 CO_2_ Clearance from Dead Space 18](#_Toc169178084)

[4.5 Modelling reduction in functional lung volume in AHRF patients 20](#_Toc169178085)

[5- AHRF Patient Matching and Optimisation 21](#_Toc169178086)

[5.1 Patient Data 21](#_Toc169178087)

[5.2 Optimisation of model parameters 21](#_Toc169178088)

[6- Patient Matching and Simulator Output Tables 23](#_Toc169178089)

[6.1 Patient Characteristics Extracted from [54, 55] 23](#_Toc169178090)

[6.2 HFNC Matching Results to Patient Data 25](#_Toc169178091)

[6.3 Simulator Output Table (HFNC Results) 27](#_Toc169178092)

[References 29](#_Toc169178093)

# ICSM Pulmonary Model

Figure S1: Diagrammatic representation of the ICSM simulator

PULMONARY CAPILLARIES

ALVEOLI

TISSUE COMPARTMENT

TISSUE CAPILLARY

ARTERY

(Oxygenated blood)

MIXED VENOUS (Deoxygenated blood)

SERIAL DEADSPACE

MOUTH

Movement of O_2_

Movement of CO_2_

Signal generator

LUNGS

Serial and Parallel Deadspace + Alveoli. Inhaled air contains deadspace gas in addition to fresh gas. Inhaled gas is warmed and saturated with water.

PULMONARY PERFUSION

Shunted and non-Shunted blood flow. O_2_ and CO_2_ equilibrate across the alveolar capillary barrier with consideration to blood gas relationships.

VENOUS BLOOD

Calculation of pH and gas tensions after tissue exposure.

TISSUES

Extraction of O_2_ and production of CO_2_ according to metabolic rate and respiratory quotient.

ARTERIAL BLOOD

Combines shunted and non- shunted blood flows and resultant blood parameters. Considers HPV and calculated pH and gas tensions.

SPONTANEOUS VENTILATION

Muscle pressure modelled as piecewise continuous function described by the amplitude (inspiratory effort), rate of respiration and respiratory cycle with an inspiration and expiration phase.

RESPIRATORY SUPPORT

Positive pressure support applied at the mouth. It is assumed the IE ratio and ventilation rate is equal to that of the spontaneous ventilation.

The model employed in this paper has been developed over the past several years and has been applied and validated on a number of different studies [1– 10]. The model is organised as a system of several components (see Figure S1), each component representing different aspects of pulmonary dynamics and blood gas transport, e.g. the transport of air in the mouth, the tidal flow in the airways, the gas exchange in the alveolar compartments and their corresponding capillary compartment, the flow of blood in the arteries, the veins, the cardiovascular system, and the gas exchange process in the peripheral tissue compartments. Each component is described as several mass conserving functions and solved as algebraic equations, obtained or approximated from the published literature, experimental data and clinical observations. These equations are solved in series in an iterative manner, so that solving one equation at current time instant $(t_{k})$ determines the values of the independent variables in the next equation. At the end of the iteration, the results of the solution of the final equations determine the independent variables of the first equation for the next iteration.

The iterative process continues for a predetermined time, *T*, representing the total simulation time, with each iteration representing a ‘time slice’ *t* of real physiological time (set to 10 ms). At the first iteration$(t_{k}, k=0)$, an initial set of independent variables are chosen based on values selected by the user. The user can alter these initial variables to investigate the response of the model or to simulate different pathophysiological conditions. Subsequent iterations ($t_{k}= t_{k-1}+t$) update the model parameters based on the equations below.

The pulmonary model consists of the mechanical ventilation equipment, anatomical and alveolar dead space, anatomical and alveolar shunts, ventilated alveolar compartments and corresponding perfused capillary compartments. The pressure differential created by the mechanical ventilator or inspiratory muscles (i.e. when modelling spontaneous breathing) drives the flow of gas through the system. The series dead space (SD) is located between the mouth and the alveolar compartments and consists of the trachea, bronchi and the bronchioles where no gas exchange occurs. Inhaled gases pass through the SD during inspiration and alveolar gases pass through the SD during expiration. In the model, an SD of volume 150 ml is split into 50 stacked layers of equal volumes ($N_{SD}$ = 50).

Any residual alveolar air in the SD at the end of expiration is re-inhaled as inspiration is initiated. This residual air is composed of gases exhaled from both perfused alveolar compartments (normal perfusion) and the parallel dead space (PD) (alveolar compartments with limited perfusion). Therefore, the size of dead space (SD and PD) can have a significant effect on the gas composition of the alveolar compartments.

The inhaled air is initially assumed to consist of five gases: oxygen (O_2_), nitrogen (N_2_), carbon dioxide (CO_2_), water vapor (H_2_O) and a 5th gas (α) used to model additives such as helium or other anaesthetic gases. During an iteration of the model, the flow (*f*) of air to or from an alveolar compartment *i* at time $t_{k}$is determined by the following equation:

$f_{i}=\frac{\left( P_{trachea}- p_{i} \right)}{\left( \text{R}_{\text{L\_aw, }i} \right)}$ $\mathrm{for} i=1,\ldots,N_{A}$ (1)

and

$f_{SD}= \sum_{i=1}^{N_{A}} f_{i}$ (2)

is the total flow, where $N_{A}$is the total number of alveolar compartments (for the results in this study, $N_{A}$ = 100). During inhalation,$f_{SD}>0$, while during exhalation $f_{SD}<0$.

In the above equations:

*R_L_aw, i_* is the lower airway resistance, and is equal to $\text{R}_{\text{B}}+\text{R}_{\text{A,}i}$ where *R_B_* is the resistance of the bronchi and bronchioles, and *R_A,i_* is the inlet resistance of the *i^th^* alveolar compartment which is defined by:

$R_{A,i}=m_{i}R_{A0}$ (3)

where $R_{A0}$ corresponds to the default bronchial inlet resistance of an alveolar compartment. $R_{A0}$ is normally distributed around the mean value of ${10}^{-7}\times N_{A}$ (the inlet resistance is higher for a model with more compartments as the volume of each compartment decreases) for a healthy lung. $m_{i}$ is a coefficient of the airway resistance, representing a dynamic change in airway resistance and is determined by the equation:

$m_{i}=\left\{ \begin{aligned} 1, t_{o,i}\leq0 \\ {10}^{15}, t_{o,i}>0 \end{aligned} \right.\text{ }\text{for}\text{ }i=1, \ldots,N_{A}$ (4)

where,

$t_{o, i}=\left\{ \begin{aligned} t_{o,i}-t , P_{trachea}\geq\text{TOP}_{\text{i}} \\ \tau_{c,i}, P_{trachea}<\text{TOP}_{\text{i}} \end{aligned}\text{ for}i=1, \ldots,N_{A} \right.$ (5)

$P_{trachea}$ is the pressure at the trachea which is calculated as:

$P_{trachea}=\left\{ \begin{aligned} P_{inlet}-\left( \frac{P_{inlet}-P_{lungs}}{R_{aw}}\times R_{{U\_}_{aw}} \right) Inhalation \\ P_{inlet}-\left( \frac{P_{lungs}-P_{inlet}}{R_{aw}}\times R_{{L\_}_{aw}} \right) Exhalation \end{aligned} \right.$ (6)

where $P_{inlet}=P_{atm}$ (in the absence of external support) (7)

$P_{lungs}= \sum_{i=1}^{N_{A}} \left( \frac{p_{i}}{\text{R}_{\text{L\_aw, }i}} \right)\times R_{L\_aw}$ (8)

$R_{L\_aw}=\frac{1}{\sum_{i=1}^{N_{A}} \left( \frac{1}{\text{R}_{\text{L\_aw, }i}} \right)}$ (9)

$R_{U\_aw}$ is the upper airway resistance which represents the nasal cavity, oral cavity and trachea and $R_{aw}$ is the total respiratory airway resistance (i.e. $R_{U\_aw}$ + $R_{L\_aw}$).

Lung pressure ($P_{lungs}$) results from the summation of the flow at each alveolar compartment multiplied by the equivalent resistance of the lower airways ($R_{L\_aw}$), i.e., from the end of the trachea to the lungs.

$p_{i}$ is the pressure in the *i*^th^ alveolar compartment. Each alveolar compartment has a unique and configurable alveolar stiffness ($S_{i}$), alveolar inlet resistance, vascular resistance, extrinsic (interstitial) pressure ($P_{ext}$), threshold opening pressure and threshold closing pressure. For the $i$^th^ compartment of *N* alveolar compartments, the pressure $p_{i}$ is determined by equation (10) at a given volume of $v_{i}$ in millilitres:

$p_{i}{(t}_{k})=P_{atm}+S_{i}\left( v_{i}{(t}_{k})-V_{c} \right)^{2}-P_{ext,i}+ P_{INSP}{(t}_{k}) for i=1,\ldots,N_{A}$ (10)

where

$S_{i}=k_{i}{N_{A}}^{2}/200000$ and $V_{c}=0.2V_{FRC}/N_{A}$ (11)

The alveolar compartments are arranged in parallel and interact with the series dead space with respect to the movement of gases. The use of the square of the difference between $v_{i}$ and $V_{c}$ causes alveolar pressure to increase at volumes below $V_{c}$, leading to exhalation and a tendency to “snap shut” (mathematical note: the pressure with respect to volume is thus a U-shaped curve) [11].

$P_{ext}$ (per alveolar unit, in cmH_2_O) represents the effective net pressure generated by the sum of the effects of factors outside each alveolus that act to distend that alveolus; positive components include the outward pull of the chest wall, and negative effects include the compressive effect of interstitial fluid within the pleural cavity. Incorporating $P_{ext}$ in the model allows us to replicate the situation of alveolar units that have less structural support or that have interstitial oedema, and thus have a greater tendency to collapse or consolidate. A negative value of $P_{ext}$ indicates a scenario where there is compression from outside the alveolus causing collapse. The parameter $S_{i}$ is a scalar that determines the intra-alveolar pressure for a given volume (with respect to a constant collapsing volume$V_{c}$) and is dependent on the parameter$k$. The units of $S_{i}$ are cmH_2_O ml^-2^. $P_{INSP}$, which represents the pressure generated by the respiratory muscles acting on the lung, is described in the next section. Finally, $V_{c}$ is defined as a “constant collapsing volume” at which the alveolus tends to empty (through Laplace effects) and represents a fundamental mechanical property of tissue and surfactant [11]. $V_{FRC}$ is the end expiratory volume of the lungs.

The effect of the three parameters on the volume–pressure relationship of the alveolar compartments can be observed in Figure S2.


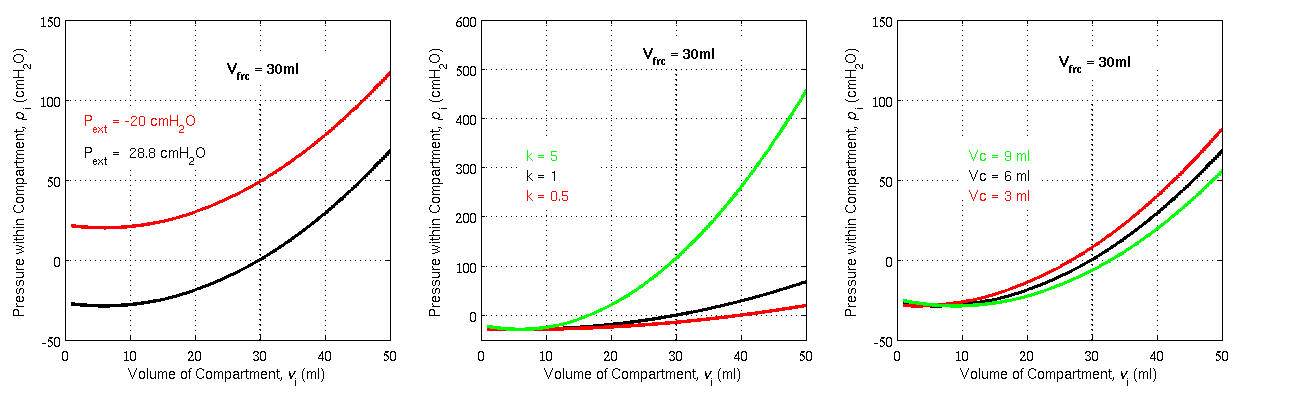


Figure S2: The effect of varying the parameters of Equation (10) on the pressure volume relationship of the model ($P_{INSP}$ = 0) and under mechanical ventilation.

The nominal values for ($P_{ext,i}$, $S_{i}$ ) have been determined such that at the end of expiration, the alveolar pressure within the compartment is also equal to zero, i.e. at a pre-set value of functional residual capacity.

We consider each of the parameters mentioned above ($P_{ext,i}$,$S_{i}$) to be different yet essential components for representing a diseased lung that affect the volume pressure relationship of the alveolar compartments. For example, for a given volume$v_{i}$, increasing$S_{i}$ increases the corresponding alveolar pressure of the alveolar compartment. When compared to another compartment with a lower$S_{i}$, a larger pressure gradient would be needed to drive air into the compartment; thus, effectively the compartment will be behaving as a stiffer lung unit.

Decreasing $P_{ext,i}$ to a negative value increases the alveolar pressure such that the pressure gradient (especially during exhaling) forces the air out of the alveolar compartment until the volume of the compartment collapses ($v_{i}$ = 0 ml). Note that, in effect, the parameters are influencing the resting volume of the compartments (when the alveolar pressure,$p_{i}$, is equal to zero).

$TOP_{i}$ is a value between 5 and 50 cmH_2_O for the $i^{th}$ alveolar compartment. A threshold opening pressure (TOP) at low lung volumes needs to be attained for a collapsed alveolar unit to open. Recruitment is a time dependent process, with different airways recruiting at different times, once the threshold opening pressure has been achieved [12], [13]. The equations within the model are solved iteratively as a discretised system. Each iteration represents a physiological time slice of *t* (10 ms). The time-dependent recruitment phenomenon is achieved in the model by the introduction of a parameter$t_{o}$. For collapsed compartments, $t_{o}$ is set to $\tau_{c}$ which represents the time it would take for collapsed alveoli to open after a threshold pressure is reached. Once $P_{trachea}\geq\text{TOP}_{\text{i }}$ is satisfied, the counter $t_{o}$ decrements during every iteration and triggers the opening of the airway ($m_{i}$= 1) as $t_{o}\leq0$. Otherwise $m_{i}$ is set to a high value (10^15^) to represent a collapsed airway.

$N_{A}$ (the number of alveolar compartments) is fixed and set by the user (i.e. they do not change during a simulation). Therefore, during a simulation, $m_{i}$, chiefly represents the relatively small changes in inlet resistance during tidal ventilation. Furthermore, $R_{B}$is also preset and fixed, and do not change during the simulation. The only change in airway resistance which is dynamic is $m_{i}$ which is dependent on the volume$v_{i}$at time${(t}_{k})$.

Finally, the pulmonary vascular resistance PVR is determined by:

| $\frac{1}{PVR}=\frac{1}{R_{V,1}}+\frac{1}{R_{V,2}}+\cdots+\frac{1}{R_{V,N_{A}}}, \text{for }i=1, \ldots,N_{A}$ | (12) |
| --- | --- |

where the resistance for each compartment $R_{V,i}$is defined as

| $R_{V,i}=\delta_{Vi}R_{V0}$ | (13) |
| --- | --- |

$R_{V0}$ is the default vascular resistance for the compartment with a value of $160\cdot N_{A}$ dynes s cm^-5^ min^-1^, and $\delta_{Vi}$ is the vascular resistance coefficient, used to implement the effect of hypoxic pulmonary vasoconstriction.

During gas movement in the SD, the fractions of gases in the layer $l$ of the SD,$F_{l,} (l =1,\ldots, N_{SD}$) are updated based on the composition of the total flow, $f_{SD}$, and the current composition of$F_{l,}$. If $f_{SD}$ ≥ 0, then air starts filling from the top layer ($l= 1$) to the bottom layer ($l= N_{SD}$); and vice versa for $f_{SD}$<0. The volume of gas $\text{x}$, in the $i^{th}$ alveolar compartment ($\text{v}_{i\text{, x}}$), is given by:

$v_{i, x}(t_{k}) = \left\{ \begin{aligned} v_{i, x}\left( t_{k-1} \right)-f_{i}\left( t_{k} \right)\cdot\frac{v_{i, x}\left( t_{k-1} \right)}{v_{i}{(t}_{k})} Exhaling \\ v_{i, x}(t_{k-1}) +f_{i}(t_{k}) \cdot F_{N_{SD}}(t_{k}) Inhaling \end{aligned} \right.$ $\mathrm{for} i=1,\ldots,N_{A}$ (14)

In (14), x is any of the five gases (O_2_, N_2_, CO_2_, H_2_O or α). The total volume of the $i^{th}$ alveolar compartment, $v_{i}$ is the sum of the volume of the five gases in the compartment.

$v_{i}(t_{k})= v_{i,O2}(t_{k})+ v_{i,N2}(t_{k})+v_{i,CO2}(t_{k})+v_{i,H2O}(t_{k})+v_{i,\alpha}(t_{k})$ (15)

For the alveolar compartments, the tension at the centre of the alveolus and at the alveolar capillary border is assumed to be equal. The respiratory system has an intrinsic response to low oxygen levels in blood which is to restrict the blood flow in the pulmonary blood vessels, known as hypoxic pulmonary vasoconstriction (HPV). This is modelled as a mathematical function, resembling the stimulus response curve suggested by Marshall [14], and is incorporated into the simulator to gradually constrict the blood vessels as a response to low alveolar oxygen tension. The atmospheric pressure is fixed at 101.3 KPa and the body temperature is fixed at 37.2°C.

At each $t_{k}$, equilibration between an alveolar compartment and the corresponding capillary compartment is achieved iteratively by moving small volumes of each gas between the compartments until the partial pressures of these gases differ by <1% across the alveolar-capillary boundary. The process includes the nonlinear movement of O_2_ and CO_2_ across the alveolar capillary membrane during equilibration.

In blood, the total O_2_ content (C_O2_) is carried in two forms, as a solution and as oxyhaemoglobin (saturated haemoglobin):

$\text{C}_{\text{O2}}(t_{k})=\text{S}_{\text{O2}}(t_{k-1})\cdot Huf\cdot\text{Hb + }\text{P}_{\text{O2}}(t_{k-1})\cdot O_{2sol}$ (16)

In this equation, S_O2_ is the haemoglobin saturation, *Huf* is the Hufner constant, $\text{Hb}$ is the haemoglobin concentration and $O_{2sol}$ is the O_2_ solubility constant. The following pressure-saturation relation, as suggested by [15] to describe the O_2_ dissociation curve, is used in this model:

${\text{S}\text{aO}}_{\text{2}}(t_{k}) =\left( \left( \left( {\text{P}\text{aO}}_{\text{2}}^{3}(t_{k-1})+150\cdot{\text{P}\text{aO}}_{\text{2}}(t_{k-1}) \right)^{-1}\times23400 \right)+1 \right)^{-1}$ (17)

${\text{S}\text{aO}}_{\text{2}}$ is the saturation of the haemoglobin in blood and ${\text{P}\text{aO}}_{\text{2}}$is the partial pressure of oxygen in the blood. As suggested by [16], ${\text{P}\text{aO}}_{\text{2}}$has been determined with appropriate correction factors in base excess BE, temperature T and pH (7.5005168 = pressure conversion factor from KPa to mmHg):

${\text{P}\text{aO}}_{\text{2}}(t_{k})=7.5006\text{168 ∙ }{\text{P}\text{aO}}_{\text{2}}(t_{k-1}) \cdot{10}^{\left[ 0.48\left( \text{pH}(t_{k-1})\text{-7.4} \right)-0.024\left( \text{T-37} \right)-0.0013\cdot\text{BE} \right]}$ (18)

The CO_2_ content of the blood (C_CO2_) is deduced from the plasma CO_2_ content (C_CO2plasma_) [17] by the following equation:

$\text{C}_{\text{CO2}}(t_{k})= \text{C}_{\text{CO2plasma}}(t_{k-1}) \cdot\left[ 1-\frac{0.0289\cdot\text{Hb}}{\left( 3.352-0.456 . {\text{S}\text{aO}}_{\text{2}}(t_{k}) \right)\cdot\left( 8.142-\text{pH}(t_{k-1}) \right)} \right]$ (19)

where ${\text{S}\text{aO}}_{\text{2}}$ is the O_2_ saturation, $\text{Hb}$ represents the haemoglobin concentration, and pH is the blood pH level. The coefficients were determined as a standardized solution to the McHardy version of Visser’s equation [18], by iteratively finding the best fit values to a given set of clinical data. The value of $\text{C}_{\text{CO2plasma}}$ is deduced using the Henderson-Hasselbach logarithmic equation for plasma C_CO2_ [19]_:_

$\text{C}_{\text{CO2plasma}}{(t}_{k})=2.226\cdot s_{CO2} \cdot{\text{P}\text{aCO}}_{\text{2}}(t_{k-1}) \left( 1+ {10}^{\left( \text{pH}(t_{k-1}) \text{ – pK'} \right)} \right)$ (20)

where $s_{CO2}$ is the plasma CO_2_ solubility coefficient and $\text{pK}\text{'}$ is the apparent pK (acid dissociation constant of the CO_2_ bicarbonate relationship). ${\text{P}\text{aCO}}_{\text{2}}$ is the partial pressure of CO_2_ in plasma and ‘2.226’ refers to the conversion factor from millimoles per litre to ml/100ml. [19] gives the equations for $s_{CO2}$ and $\text{pK}\text{'}$ as:

$s_{CO2}\text{= 0.0307 + 0.0057 ∙ }\left( 37-\text{T} \right)\text{ + 0.00002}{\cdot\left( 37-\text{T} \right)}^{2}$ (21)

$\text{pK}\text{' = 6.086 +0.042 ∙ (7.4 - pH}(t_{k-1}) \text{) + }\left( 38-\text{T} \right) \cdot\left( 0.00472+\left( 0.00139- \left( 7.4-\text{pH}(t_{k-1}) \right) \right) \right)$ (22)

${\text{P}\text{aCO}}_{\text{2}}\left( t_{k} \right)$is determined by incorporating the standard Henry’s law and the $s_{CO2}$(the CO_2_ solubility coefficient above). For pH calculation, the Henderson Hasselbach and the Van Slyke equation [20] are combined. Below is the derivation of the relevant equation. The Henderson-Hasselbach equation (governed by the mass action equation (acid dissociation)) states that:

$\text{pH = }\text{pK}\text{ + log }\left( \frac{bicarbonateconcentration}{carbonicacidconcentration} \right)$ (23)

Substituting pK=6.1 (under normal conditions) and the denominator $(0.225 \cdot{\text{P}\text{aCO}}_{\text{2}})$ (acid concentration being a function of CO_2_ solubility constant 0.225 and PaCO_2_ (in kPa)) gives:

$\text{pH}{(t}_{k})\text{ = 6.1 + }\log\left( \frac{\text{HCO}_{\text{3}}(t_{k-1})}{0.225 \cdot{\text{P}\text{aCO}}_{\text{2}(t_{k})}} \right)$ (24)

For a given pH, base excess (BE), and haemoglobin content (Hb), HCO_3_ is calculated using the Van-Slyke equation, as given by [20]:

$\text{HCO}_{\text{3}}{(t}_{k})= \left( \left( 2.3 \times\text{Hb}+7.7 \right)\times\left( \text{pH}(t_{k})-7.4 \right) \right)+ \frac{\text{BE}}{\left( 1-0.023 \times\text{Hb} \right)}+ 24.4$ (25)

The capillary blood is mixed with arterial blood using the equation below which considers the anatomical shunt ($Sh)$ with the venous blood content of gas x ($\text{C}_{\text{v, x}})$, the non-shunted blood content from the pulmonary capillaries ($\text{C}_{\text{cap, x}}$), arterial blood content $\text{(}\text{C}_{\text{a, x}})$, the arterial volume $\text{(v}_{a})$ and the cardiac output (CO).

$\text{C}_{\text{a, x}}(t_{k})=\frac{\text{CO}(t_{k})\text{ ∙ }\left( Sh \cdot\text{C}_{\text{v, x}}(t_{k}) + \left( 1-Sh \right) \cdot\text{C}_{\text{cap, x}}(t_{k}) \right)+ \text{C}_{\text{a, x}}(t_{k})\cdot\left( \text{v}_{a}(t_{k})- \text{CO}(t_{k}) \right)}{\text{v}_{a}(t_{k})}$ (26)

The peripheral tissue model consists of a single tissue compartment, acting between the peripheral capillary and the active tissue (undergoing respiration to produce energy). The consumed O_2_ (V_O2_) is removed, and the produced CO_2_ (V_CO2_) is added to this tissue compartment. As per the alveolar equilibration, peripheral capillary gas partial pressures reach equilibrium with the tissue compartment partial pressures, with respect to the nonlinear movement of O_2_ and CO_2_. Metabolic production of acids, other than carbonic acid via CO_2_ production, is not modelled. After peripheral tissue equilibration of gases, the venous calculations of partial pressures, concentrations and pH calculations are done using comparable equations to those above.

A simple equation of renal compensation for acid base disturbance is incorporated. The base excess (BE) of blood under normal conditions is zero. BE increases by 0.1 per time slice if pH falls below 7.36 (to compensate for acidosis) and decreases by 0.1 per time slice if pH rises above 7.4 (alkalosis).

The net effect of these components of the simulation is that the defining, clinical features of acute respiratory disease may be observed in the model: alveolar gas-trapping (with intrinsic PEEP), collapse-reopening of alveoli (with gradual reabsorption of trapped gas if re-opening does not occur), limitation of expiratory flow etc.

# Modelling Spontaneous Breathing

The pressure at the airway opening (i.e. nares or mouth) is relatively constant at atmospheric pressure, while the alveolar pressure value varies during inspiration and expiration. The active contraction of the respiratory muscles during inspiration generates a negative alveolar pressure relative to the atmospheric pressure. The variable $P_{INSP}$, which represents the pressure generated by the respiratory muscles acting on the lung, is modelled as a piecewise function as described in [21] and adapted from [22]. The function consists of a parabolic profile during the inspiration phase of the respiratory cycle, representing the progressive increase in pressure exerted by the respiratory muscles, followed by an exponential profile during the expiration phase of the respiratory cycle, characterizing the passive relaxation of the muscles. During a single respiratory cycle, $P_{INSP}$ at time $t_{k}$, is calculated as

$P_{INSP}\left( t_{k} \right)= \left\{ \begin{aligned} \frac{-P_{MIN}}{T_{I}.T_{E}}.{t_{k}}^{2}+\frac{P_{MIN}.T}{T_{I}.T_{E}}.t_{k} t_{k} \in\left\lfloor0,T_{I} \right\rfloor\\ \frac{P_{MIN}}{1-e^{-\frac{T_{E}}{\tau}}}.\left( {e^{-\frac{\left( {{(t}_{k}-T}_{I} \right)}{\tau}}-e}^{- \frac{T_{E}}{\tau}} \right) t_{k} \in\left\lfloor T_{I}, T \right\rfloor\end{aligned} \right.$ (27)

$P_{INSP}$ decreases from zero to its minimum end-inspiratory value ($P_{MIN}$) (i.e., maximum effort) during inspiration and returns to zero at end of expiration. $T$ is calculated from the set respiratory rate, RR, ($T$ = 60/RR). $T_{I}$ and $T_{E}$ are the duration of inspiration and expiration, such that ($T$ = $T_{I}+ T_{E}$). $T_{I}$ is calculated from ($T_{I}=T*DC)$ where $DC$ is the duty cycle (in the range of 0.25 to 0.5). $\tau$ is the time constant of the expiratory profile and is set to Te/RR.

The resulting alveolar pressure $p_{i}$ depends on $P_{INSP}$, as well as on the pressures of the gases within the compartment, the stiffness of the compartment ($S_{i}$), and parameter $P_{ext}$ which represents extrinsic pressures acting on the compartment.

# Calculating Lung Mechanics Parameters

The following subsections list the methodology for the calculation of each of the key indices of lung injury presented in this study.

## Compliance

The respiratory system compliance ($C_{rs}$) is calculated using:

$C_{rs}=\frac{VT}{\Delta P_{lungs}}$ (28)

where $VT$is the tidal volume, and $\Delta P_{lungs}$ is the pressure swing between peak lung pressure and minimum lung pressure. Note that this equation gives the respiratory system compliance, and not the lung compliance, as we are applying $P_{INSP}$ and $P_{ext}$ directly to the alveolar compartments.

To calculate the lung compliance ($C_{L}$) we use the elastance equation:

$\frac{1}{C_{L}}=\frac{1}{C_{rs}}-\frac{1}{C_{cw}}$ (29)

where $C_{cw}$ is the chest wall compliance, estimated to be 4% of the vital capacity in ml [23]:

$C_{cw}=0.04 \times VC$ (30)

Vital capacity is estimated using the following equations [24]:

$\left\{ \begin{aligned} {VC}_{male}(ml)=27.63-\left( 0.112\times Age(years) \right)\times Height(cm) \\ {VC}_{female}(ml)=21.78-\left( 0.101\times Age\left( years \right) \right)\times Height(cm) \end{aligned} \right.$ (31)

## Transpulmonary Pressure

The transpulmonary pressure ($P_{L}$) was calculated as:

$P_{L}=\frac{\Delta V}{C_{L}}$ (32)

where $C_{L}$ is the dynamic lung compliance, and $\Delta V$ is the difference between the current lung volume and the unstressed lung volume (the residual volume, $V_{RV}$).

The residual volume was estimated for each patient using the following equations [25]:

$\left\{ \begin{aligned} V_{RV,male}(L)=\left( 1.31\times Height(m) \right)+\left( 0.022\times Age(year) \right)-1.23 \\ V_{RV,female}(L)=\left( 1.81\times Height(m) \right)+\left( 0.016\times Age(year) \right)-2 \end{aligned} \right.$ (33)

## Pleural Pressure

The pleural pressure ($P_{pl}$) was derived from the transpulmonary ($P_{L}$) and lung pressures:

$P_{pl}=P_{lungs}-P_{L}$ (34)

## Baseline End Expiratory Lung Volume

To calculate the total strain being applied to the lungs, we first need to determine a baseline end-expiratory lung volume against which we will compare the change in inspired lung volume. To estimate this, respiratory rate was set to 14 bpm and muscle pressure adjusted to achieve a tidal volume set to 7 ml/kg of predicted body (to emulate quiet breathing). The end-expiratory lung volume ($EELV$) was then recorded and used as $EELV_{Baseline}$.

## Total Lung Strain

The total lung strain was calculated as the sum of the static and dynamic strains across the lung [26]. The dynamic and static strains are calculated as follows:

$Dynamic Strain=\frac{VT}{EELV_{Baseline}}$ (35)

$Static Strain=\frac{EELV-EELV_{Baseline}}{EELV_{Baseline}}$ (36)

$Total Strain=Dynamic Strain+Static Strain$ (37)

## Total Lung Stress

The total stress applied to the lungs is, by definition, the stress (pressure) applied to the lungs attributed to the difference between the unstressed volume of the lung, $V_{RV}$, and the current volume of the lung. Therefore, the total lung stress is equal to the transpulmonary pressure swing ($\Delta P_{L}$) [27].

$\Delta P_{L}=P_{L,max}-P_{L,min}$ (38)

## Mechanical Power

The energy applied to the lungs per breath is equal to the area enclosed by the pressure-volume curve. To determine the power, we multiply this area by the respiratory rate and apply a unit conversion factor, as described in [28]:

$Power=\left( 0.5\times VT\times\Delta P_{L} \right)\times RR\times0.0000980665$ (39)

Here, $VT$ is tidal volume in ml, $\Delta P_{L}$ represents transpulmonary pressure swing in cmH_2_O, and $RR$ is respiratory rate in bpm. Consequently, $Power$ is calculated in J/min.

## Driving Pressure

The concept of driving pressure ($DP$), whilst being well defined in invasive mechanical ventilation as the difference between plateau pressure ($P_{plat}$) and $PEEP$, is a more challenging concept in spontaneous ventilation due to the absence of a satisfactory $P_{plat}$. It has been proposed that $DP$ can be approximated in spontaneously breathing patients receiving non-invasive respiratory support by using an inspiratory hold to get an estimate of $P_{plat}$ [29], but this is still an approximation of the true $\Delta P$ which is generating the inspiratory flow.

Within the model, we are able to calculate the change in lung pressure which occurs during the inspiratory phase as a result of the support and the patient’s inspiratory efforts, allowing an accurate calculation of the pressure which is distending the lung.

In spontaneous breathing, $DP$ can be calculated as the difference between the end-expiratory lung pressure ($EELP$) and the minimum lung pressure ($P_{lungs,min}$), which represents the pressure differential driving the flow:

$DP= EELP-P_{lungs,min}$ (40)

# Modelling High Flow Nasal Cannula Therapy

To accurately recreate the effects of high flow nasal cannula (HNFC) therapy, we need to consider the differing effects on pressure dynamics during both the inspiratory and expiratory phases. When HFNC is applied, the pressure at the nares increases, primarily attributed to the HFNC-induced pressure.

To recreate this phenomenon in our model, we calculate the corresponding increase in pressure by multiplying the HFNC flow by the subject’s total airway resistance (R_aw_) (Eq. 28). The modelled R_aw_ comprises two main respiratory airway resistances which are located in series. The first resistance, the upper airways (R_U_ aw_), represents the nasal cavity, oral cavity, and trachea whilst the second resistance, the lower airways (R_L_aw_), represents the bronchi and the bronchioles, and the inlet resistances for multiple alveoli which make up the lungs, placed in parallel.

To simulate the effects of High Flow Nasal Cannula (HFNC) therapy, we model appropriate changes in pressure dynamics during the inspiratory and expiratory phases. The flow from HFNC increases pressure at the nares, calculated as the product of the flow rate and the subject's total airway resistance (R_aw_). Hence, Eq. 7 is updated accordingly to represent the HFNC induced elevated pressure at the nares:

$P_{inlet}=P_{atm}+\left( flow rate\times R_{aw} \right)$ (28)

However, during HFNC, not all the flow delivered from the device (set flow rate) takes part in producing a positive pressure at the upper airway. We consider this non-effective flow (i.e. flow that does not contribute to creating a positive pressure) as leakage. To reflect this, we modify the updated equation for $P_{inlet}$ as follows:

$P_{inlet}=P_{atm}+\left( effective flow rate\times R_{aw} \right)$ (29)

Under HFNC, mean airway pressure (mP_aw_) is mean tracheal pressure throughout one complete breathing cycle (Eq. 30).and positive end expiratory pressure (PEEP) represents the end expiratory tracheal pressure:

${mP}_{aw}=mean (P_{trachea})$ (30)

## Experimental studies measured airway pressures

Previous experimental studies, which measured airway pressures, were identified through an electronic search of the PubMed database using the following search terms: "(high flow nasal) AND ((airway pressure) OR (pharyngeal pressure)) AND ((healthy subjects) OR (healthy patients)) NOT (children)." The search was conducted on 17^th^ March 2024, with no language or date of publication restrictions applied.

Table S1: Summary of the studies measured the airway pressure produced by HFNC at different flow rates

| **Study** | **No. of subjects** | **Condition** | **Measured pressure** | **Flow rate** (L/min) | **Mouth Closed Pressure** (cmH_2_O) | **Mouth Open Pressure** (cmH_2_O) |
| --- | --- | --- | --- | --- | --- | --- |
| Groves et al. 2007 [37] | 10 (5Males/5Females) | Healthy | Expiratory pressures | 10 20 40 60 | 1.7 [1.2-2.3]* 2.9 [2.2-3.7] 5.5 [4.1-7.2] 7.4 [5.4-8.8] | 0.7 [0.6-0.9] 1.4 [1.3-1.8] 2.3 [2.0-2.5] 3.1 [2.4-3.1] |
| Parke et al. 2009 [38] | 15 | Surgery | Mean airway pressure | 35 | 2.7 (1.04)** | 1.2 (0.76) |
| Parke et al. 2011 [39] | 12 (9Males/3Females) | Surgery | Mean airway pressure | 30 40 50 | 1.93 (1.25) 2.58 (1.54) 3.31 (1.05) | 1.03 (0.67) 1.30 (0.80) 1.73 (0.82) |
| Corley et al. 2011 [32] | 20 (15Males/5Females) | Healthy | Mean airway pressure | 35 to 50 | 2.7 (1.2) | NA |
| **Ritchie et al. 2011** [40] | **10 (8Males/2Females)** | **Healthy** | **Mean airway pressure** | **10 20 30 40 50** | **0.5 (NA) 1.2 (NA) 2.5 (NA) 4 (NA) 5.1 (NA)** | **0.1 (NA) 0.6 (NA) 0.1 (NA) 0.8 (NA) 1.1 (NA)** |
| Braunlich et al. 2013 [33] | 16 (6Males/10Females) | Healthy | Mean airway pressure | 20 | 1 (NA) | NA |
| Parke et al. 2013 [41] | 15 (14Males/1Female) | Surgery | Mean airway pressure | 30 40 50 | 1.52 (0.6) 2.21 (0.8) 3.10 (1.2) | NA |
| **Parke et al. 2015** [42] | **15 (7Males/8Females)** | **Healthy** | **Mean airway pressure** | **30 40 50 60 70 80 90 100** | **2.71 (0.74) 3.79 (0.84) 4.95 (1.09) 6.12 (1.37) 7.69 (1.46) 9.00 (1.89) 10.05 (2.11) 11.88 (2.73)** | **NA** |
| Okuda et al. 2017 [30] | 10 (10Males/0Female) | Healthy | ∆ Oesophageal pressure | 30 50 | 6.9 (2.9) 7.48 (3.1) | NA |
| Komatsu et al. 2019 [43] | 18 (9Males/9Females) | Healthy | Mean expiratory pressure | 40 | NA | 2.09 [1.6-4.0] 3.35 [2.7–3.8]^†^ |
| Garofalo et al. 2019 [44] | 14 (7Males/7Females) | Healthy | ∆ end-expiratory pressure | 30 40 50 | 2.8 (NA) 5.8 (NA) 7.7 (NA) | 0.9 (NA) 1.7 (NA) 2.3 (NA) |
| Yao et al. 2021 [45] | 44 | Healthy | end-expiratory pressure | 20 25 30 35 40 45 50 55 60 | 2.5 (NA) 3.2 (NA) 3.7 (NA) 4.3 (NA) 4.6 (NA) 5 (NA) 5.3 (NA) 5.5 (NA) 5.6 (NA) | NA |
| **Miyazaki et al. 2021** [46] | **15 (8Males/7Females)** | **Healthy** | **Mean airway pressure** | **20 30 40** | **1.09 (0.60) 2.27 (1.10) 3.51 (1.43)** | **0.31 (0.56) 0.58 (0.65) 1.22 (0.83)** |
| **Vieira et al. 2022** [35] | **10 (8Males/2Females)** | **Healthy** | **Mean airway pressure** | **20 40 60** | **0.94 [0.6-1.2] 3.44 [2.6-4.1] 6.89 [4.7-7.4]** | **0.18 [0.0-0.3] 0.54 [0.4-0.8] 0.75 [0.5-1.1]** |
| Zhao et al. 2023 [47] | 35 (16Males/19Females) | Healthy | end-expiratory pressure | 20 30 40 50 60 | 2.08 (1.75) 3.12 (2.06) 4.92 (2.53) 6.21 (2.93) 7.18 (4.18) | NA |

* Ranges in brackets; ** Standard Deviation in parentheses; † Mouth Open with Face Mask; NA: Not reported

In addition to the database search, a supplementary exploration was conducted on Google Scholar to identify any potential additional studies that might have been omitted from the initial search. A summary of the results of these experimental studies is presented in Table S1.

## Creating a Virtual Cohort of Healthy Subjects

Table S2: characteristics of ten virtual subjects

|  | **Subject 1** | **Subject 2** | **Subject 3** | **Subject 4** | **Subject 5** | **Subject 6** | **Subject 7** | **Subject 8** | **Subject 9** | **Subject 10** |
| --- | --- | --- | --- | --- | --- | --- | --- | --- | --- | --- |
| **Sex** | M | M | M | F | F | F | M | M | F | F |
| **Age (year)** | 22 | 34 | 60 | 33 | 44 | 50 | 25 | 23 | 23 | 21 |
| **Height (cm)** | 185 | 178 | 173 | 170 | 164 | 159 | 180 | 175 | 160 | 155 |
| **Weight (kg)** | 75 | 80 | 85 | 76 | 65 | 60 | 83 | 77 | 69 | 61 |
| **BMI** | 21.9 | 25.2 | 28.4 | 26.5 | 24.2 | 23.7 | 25.6 | 25.1 | 27.0 | 25.4 |
| **CO (L/min)** | 5.9 | 5.6 | 5.5 | 5.4 | 5.2 | 5.0 | 5.7 | 5.5 | 5.0 | 4.9 |
| **VC (ml)** | 4656 | 4258 | 3627 | 3131 | 2843 | 2660 | 4469 | 4385 | 3113 | 3047 |
| **FRC (ml)** | 3252 | 3241 | 3351 | 2845 | 2622 | 2468 | 3264 | 3229 | 2665 | 2606 |
| **R_aw_ (cmH_2_O.s/L)** | 3.8 | 4.3 | 5 | 5.8 | 6.5 | 7.4 | 4 | 4.8 | 5.9 | 6.1 |
| **C_CW_ (ml/cmH_2_O)** | 186 | 170 | 145 | 125 | 114 | 106 | 179 | 175 | 125 | 122 |
| **C_L_ (ml/cmH_2_O)** | 215 | 178 | 161 | 140 | 126 | 112 | 209 | 192 | 138 | 129 |
| **C_RS_ (ml/cmH_2_O)** | 100 | 87 | 76 | 66 | 60 | 55 | 96 | 92 | 66 | 63 |
| **VT (ml)** | 520 | 468 | 463 | 456 | 382 | 345 | 505 | 459 | 390 | 368 |
| **I:E** | 1:2 | 1:2 | 1:2 | 1:2 | 1:2 | 1:2 | 1:2 | 1:2 | 1:2 | 1:2 |
| **RR (bpm)** | 13 | 14 | 15 | 14 | 15 | 16 | 13 | 13 | 14 | 14 |
| **P/F ratio (mmHg)** | 474 | 474 | 469 | 472 | 477 | 477 | 465 | 458 | 455 | 464 |
| **PaO_2_ (mmHg)** | 99.5 | 99.5 | 98.4 | 99.2 | 100.3 | 100.1 | 97.7 | 96.2 | 95.6 | 97.5 |
| **SaO_2_ (%)** | 98 | 98 | 98 | 98 | 98 | 98 | 98 | 98 | 97 | 98 |
| **PaCO_2_ (mmHg)** | 36.1 | 35.7 | 36.2 | 35.8 | 35.7 | 36.2 | 37.5 | 39.6 | 40.7 | 39.0 |
| **pH** | 7.45 | 7.45 | 7.45 | 7.45 | 7.45 | 7.44 | 7.43 | 7.41 | 7.4 | 7.42 |
| **Total strain** | 0.16 | 0.15 | 0.14 | 0.16 | 0.15 | 0.14 | 0.15 | 0.14 | 0.15 | 0.14 |
| **Stress (∆P_L_) (cmH_2_O)** | 2.4 | 2.6 | 2.9 | 3.3 | 3.0 | 3.1 | 2.4 | 2.4 | 2.8 | 2.8 |
| **Power (J/min)** | 0.8 | 0.9 | 1.0 | 1.0 | 0.9 | 0.8 | 0.8 | 0.7 | 0.8 | 0.7 |
| **∆P_pl_ (cmH_2_O)** | 5.0 | 5.3 | 6.1 | 6.9 | 6.4 | 6.5 | 5.1 | 4.9 | 5.9 | 5.9 |
| CO – Cardiac output \| VC – Vital capacity \| FRC – Functional residual capacity \| R_aw_ – Total airway resistance \| C_CW_ – Chest-wall compliance \| C_L_ – Lungs compliance \| C_RS_ – Respiratory system compliance \| VT – Tidal volume \| I:E – Inhalation to Exhalation ratio \| RR – Respiratory rate \| ∆P_L_ – Transpulmonary pressure swing \| ∆P_pl_ – Pleural pressure swing | | | | | | | | | | |

We created a cohort of ten healthy subjects to model HFNC therapy and to estimate the leakage using this therapy. Detailed information regarding the cohort is presented in Table S2. Healthy lung physiology and respiratory effort are simulated in all cases, with compliance and airway resistance values varying within normal ranges.

Based on data from the literature, we simulated reductions in respiratory rate under HFNC therapy of 3.5, 4, and 4.5 bpm for flow rates of 10-40, 40-70, and >70 L/min, respectively [30– 36]. Moreover, a correction factor ($\alpha_{R}=1.1$) was applied to R_L_aw_ in order to model the increase in airway resistance observed at flow rates used in HFNC therapy [30, 35]. For calculating R_U_aw_, the fanning friction factor for turbulent flow was employed to take into account the effect of velocity within the upper airway, enabling a more comprehensive representation of the dynamic airflow behaviour during HFNC therapy.

## Estimating leakage by calibrating the model to experimental data

We chose four experimental studies to estimate the gas flow leakage (i.e. the amount of gas that does not impact the pressure field of the subject) and calibrate the model. The selection criteria for our study were experimental investigations (i) involving the application of HFNC therapy on healthy adult subjects (excluding post- or pre-operative cases), (ii) who were breathing under normal conditions, and (iii) reporting mean airway pressures at multiple flow rates. The bold rows in Table S1 identify the final selected studies used for the estimation of leakage in this study [35, 40, 42, 46].

Table S3: Average mP_aw_, effective flow, and estimated leakage at different set flow rates across virtual patients

|  | **Set Flow rate (L/min)** | **Average mP_aw_  (cm H_2_O)** | | **Effective Flow (L/min)** | | **Estimated Leakage (%)** | | |
| --- | --- | --- | --- | --- | --- | --- | --- | --- |
|  |  | **MC** | **MO** | **MC** | **MO** | **MC** | **MO** | |
| **Study1** [40] | **20** | 1.2 | 0.6 | 12 | 6.0 | 40 | 70.0 | |
|  | **30** | 2.5 | 0.1 | 24 | 1 | 20 | 96.67 | |
|  | **40** | 4 | 0.8 | 36 | 8 | 10 | 80 | |
|  | **50** | 5.1 | 1.1 | 46 | 11 | 8 | 78 | |
| **Study2** [42] | **30** | 2.71 | - | 25 | - | 16.67 | - | |
|  | **40** | 3.79 | - | 34 | - | 15 | - | |
|  | **50** | 4.94 | - | 44 | - | 12 | - | |
|  | **60** | 6.12 | - | 54 | - | 10 | - | |
|  | **70** | 7.69 | - | 66 | - | 5.71 | - | |
|  | **80** | 9 | - | 76 | - | 5 | - | |
|  | **90** | 10.05 | - | 83 | - | 7.78 | - | |
|  | **100** | 11.88 | - | 95 | - | 5 | - | |
| **Study3** [46] | **20** | 1.09 | 0.31 | 11 | 3 | 45 | 85 | |
|  | **30** | 2.27 | 0.58 | 22 | 6 | 26.67 | 80 | |
|  | **40** | 3.51 | 1.22 | 32 | 12 | 20 | 70 | |
| **Study4** [35] | **20** | 0.94 | 0.18 | 10 | 2 | 50 | 90 | |
|  | **40** | 3.44 | 0.54 | 32 | 6 | 20 | 87.5 | |
|  | **60** | 6.89 | 0.75 | 58 | 8 | 3.33 | 90 | |
| MC: Mouth closed \| MO: Mouth open \| mP_aw_: Mean airway pressure | | | | | | | |  |

To estimate the gas flow leakage, we conducted flow titrations for all our ten virtual subjects, by adjusting the effective flow rate (i.e., the flow delivered within the simulator) until the average of mean airway pressure (*mP_aw_*) across all ten subjects was equal to the average of mean airway pressure reported in each of the experimental studies at each specific set flow rate. Then the percentage flow leakage was calculated by dividing the flow loss by the set flow rate (Eq. 31):

$Estimated Leakage \left( \% \right)=\frac{set flow rate-effective flow rate}{set flow rate}\times100$ (31)

Table S3 shows the calculated effective flow rates and corresponding estimated leakage values for each of the four studies for both closed and open mouth conditions at each set flow rate. Although the total amount of gas leakage fluctuates, the gas leakage as a percentage of the set flow rate decreases at higher flow rates. With the mouth closed, the estimated leakage ranged from 17% to 27% at 30 L/min, from 10% to 20% at 40 L/min, from 8% to 12% at 50 L/min, and from 3.3% to 10% at 60 L/min. Only Study2 administered set flow rates higher than 60 L/min – at these higher flow rates the estimated percentage leakage continued to reduce gradually, reaching 5% at a set flow rate of 100 L/min. Subjects receiving HFNC therapy with their mouths open had much higher gas flow leakage at all flow rates, with values ranging from 70% to 97%.

## CO_2_ Clearance from Dead Space

The model consists of a series dead space volume located between the inlet of the respiratory system and the alveolar compartments. This dead space is simulated as a series of stacked, rigid laminae (N_SD_ = 50), each with equal volume. The total volume of the series dead space is set to 150 ml [48, 49], with each lamina, j, containing a known fraction of the inhaled or exhaled gases (see Figure S3).

Observations from experimental studies [50– 52] indicate that the majority of dead space clearance by HFNC occurs at the end of exhalation and the beginning of inhalation. Thus, the initial 150 ml (the dead space volume) of inhalation and the final 150 ml of exhalation are earmarked for clearance. Subsequently, the volume of fresh air entering the airways in each time slice (10 ms) was determined, and this volume was divided by the volume of each layer (3 ml in this study) to identify the corresponding layers of dead space washed. The inspired gas was then blended with the content of the identified layers, replacing the layer content with the mixed gas.

$N_{washed\_layers} = \frac{HFNC flow rate\times time slice}{V_{lam}}$ (31)

$f_{new\_SD,j}^{x}= \omega\times f_{inspired}^{x}+\left( 1-\omega\right)\times f_{old_{SD},j}^{x} , j<N_{washed\_layers}$ (32)

where $f_{SD,j}^{x}$ is the fraction of gas $x$ in lamina $j$ and $\omega$ (here 0.8) is the mixing factor which determines the proportion of gas mixing during washout.

To facilitate gas mixing within the dead space, a dead space gas mixing module [53] was employed with a mixing parameter (σ). This parameter allows for varying degrees of mixing between adjacent layers, with σ = 1 indicating complete gas mixing and σ = 0 indicating no mixing. The mixing factor (σ) was determined based on the clearance half-time reported in [51], representing the extent of mixing within the dead space. To determine this factor, spontaneous breathing simulations were conducted without any support, and at the end of exhalation, the end-tidal gas content in the dead space was utilized. Subsequently, HFNC was administered, and the washout and dead space gas mixing module were implemented, resulting in clearance half-times consistent with those observed in the experiment. The resulting mixing factor was determined to be 0.1.

$f_{SD,j}^{x}=\left( (1-\sigma\right)*f_{SD,j}^{x})+\sigma*f_{SD,j+1}^{x} , j<N_{SD}$ (33)

where $f_{SD,j}^{x}$ is the fraction of gas $x$ in lamina $j$ and $f_{SD,j+1}^{x}$is the fraction of gas $x$ in the next lamina.

Figure S3: Diagram of gas movement during inhalation in serial dead space

## Modelling reduction in functional lung volume in AHRF patients

To simulate alveolar collapse/consolidation (i.e., compartments with no participation in ventilation), in the model the inlet resistance of a specified number of the $N_{A}=$100 alveolar compartments is increased to a large value to preclude any air flow to the alveolus$.$ Specifically, the coefficient of the airway resistance ($m_{i}$) in equation (3) is set to 10^15^.

In this part of the investigation, all other parameters pertaining to the subjects including FiO_2_ were left unaltered, ensuring that the results are focused exclusively on elucidating the influence of the size of the functional lung volume on the pressures generated by HFNC.

# AHRF Patient Matching and Optimisation

## Patient Data

Patient data was collected from two studies [54, 55] detailing the observations of 60 non-COVID-19 patients, experiencing moderate-to-severe Acute Hypoxemic Respiratory Failure (AHRF) under HFNC therapy. These studies were conducted at a Respiratory Intensive Care Unit (ICU) at the University Hospital of Modena, Italy, from 2016 to 2021. The data used for patient matching is displayed in Table S4.

Table S4: Clinical data which is used to match patients

| **Clinical Data** | **Unit** | **Use** |
| --- | --- | --- |
| Age | Years | Estimation of residual volume and chest wall compliance |
| Height | Meters | Estimation of residual volume and chest wall compliance |
| Gender |  | Estimation of residual volume and chest wall compliance |
| Weight | Kilograms | Direct model input |
| Fraction of inspired oxygen | % | Direct model input |
| Respiratory rate | Breaths/minute | Direct model input |
| HFNC flow rate | Litres/minute | Direct model input (used during HFNC only) |

## Optimisation of model parameters

Table S5: model parameters and their allowed range of variation in optimization process

| **Parameter** | **Number** | **Range** | **Unit** |
| --- | --- | --- | --- |
| P_ext_ | 100 | [-70, 50] | cmH_2_O |
| k | 100 | [-5, 1.5] |  |
| RQ | 1 | [0.6, 1] |  |
| VO_2,factor_ | 1 | [2, 20] | ml.min^-1^ |
| Hb | 1 | [80, 180] | g.L^-1^ |
| BE | 1 | [-5, 8] | mEq L^-1^ |
| CO_factor_ | 1 | [0.85, 1.15] |  |
| R_B_ | 1 | [0.0002, 0.0015] | KPa.min.ml^-1^ |
| P_min_ | 1 | [5, 60] | cmH_2_O |
| DC | 1 | [0.25, 0.5] |  |

Further matching is conducted by the careful calibration of the model parameters which govern the system functionality. This is done by the minimisation of a user defined cost function using a genetic algorithm (GA). The optimization problem for model matching aims to minimize the variance between the model predictions and the actual measurements from the patient. The model parameters optimized during this process include extrinsic pressure (P_ext,i_) and stiffness coefficient ($k_{i}$) for each alveolar compartment, as well as values for Respiratory Quotient (RQ), Oxygen Consumption factor (VO_2,factor_), haemoglobin concentration (Hb), Base Excess (BE), Cardiac Output factor (CO_factor_), resistance of the bronchi and bronchioles (R_B_), minimum muscle pressure (P_min_), and duty cycle (DC). Table S5 shows the allowed range of variation for these model parameters during the optimisation.

VO_2_ and CO are calculated as follows:

${VO}_{2}={VO}_{2,factor}\times weight (Kg)$ (34)

$CO={CO}_{factor}\times(235\times{weight(Kg)}^{0.71})$ (35)

The optimization problem is formulated to determine the configuration of model parameters (x) that minimises the difference between the model outputs ($\hat{Y}$) and the patient data ($Y$). The cost function ($J$), representing this error is defined as:

$\min_{x} J=\sqrt{\sum_{i=1}^{4} {w_{i}\left( \frac{\hat{Y}_{i}-Y_{i}}{Y_{i}} \right)}^{2}}$ (36)

The data incorporated into the cost function ($Y)$ and their respective weights in percent ($w_{i}$) in the optimisation cost function are detailed in Table S6.

Table S6: Data used within cost function (Y)

| **Data** | **Unit** | **Optimisation Weight (%)** |
| --- | --- | --- |
| PaO_2_ | mmHg | 30 |
| PaCO_2_ | mmHg | 30 |
| Tidal esophageal pressure swing | cmH_2_O | 20 |
| Tidal Volume | ml | 20 |

# Patient Matching and Simulator Output Tables

## Patient Characteristics Extracted from [54, 55]

Table S7:Patient characteristics

| **Patient ID** | **Diagnosis 0= pneumonia, 1= ARDS, 2= sepsis/other** | **Age (Years)** | **Sex**  **F = 0, M =1** | **Weight (Kg)** | **Height (m)** | **Residual Volume (ml)** |
| --- | --- | --- | --- | --- | --- | --- |
| 1 | 1 | 81 | 1 | 52.0 | 1.46 | 2464.6 |
| 2 | 1 | 81 | 1 | 94.5 | 1.81 | 2923.1 |
| 3 | 2 | 71 | 0 | 68.0 | 1.79 | 2375.9 |
| 4 | 1 | 46 | 1 | 75.5 | 1.80 | 2140 |
| 5 | 0 | 72 | 0 | 114.0 | 1.90 | 2591 |
| 6 | 2 | 81 | 1 | 64.0 | 1.45 | 2451.5 |
| 7 | 1 | 68 | 0 | 77.0 | 1.60 | 1984 |
| 8 | 1 | 49 | 1 | 84.5 | 1.83 | 2245.3 |
| 9 | 0 | 93 | 1 | 57.0 | 1.68 | 3016.8 |
| 10 | 1 | 81 | 1 | 61.0 | 1.69 | 2765.9 |
| 11 | 1 | 69 | 1 | 92.0 | 1.70 | 2515 |
| 12 | 1 | 68 | 1 | 113.0 | 1.88 | 2728.8 |
| 13 | 0 | 67 | 1 | 104.0 | 1.85 | 2667.5 |
| 14 | 0 | 71 | 0 | 58.0 | 1.62 | 2068.2 |
| 15 | 1 | 62 | 0 | 83.0 | 1.89 | 2412.9 |
| 16 | 1 | 79 | 1 | 89.5 | 1.94 | 3049.4 |
| 17 | 0 | 71 | 1 | 86.0 | 1.79 | 2676.9 |
| 18 | 0 | 63 | 1 | 95.0 | 1.69 | 2369.9 |
| 19 | 1 | 84 | 0 | 84.0 | 1.82 | 2638.2 |
| 20 | 1 | 81 | 1 | 80.0 | 1.61 | 2661.1 |
| 21 | 0 | 73 | 0 | 114.0 | 1.83 | 2480.3 |
| 22 | 0 | 63 | 1 | 94.5 | 1.89 | 2631.9 |
| 23 | 0 | 52 | 0 | 96.0 | 1.71 | 1927.1 |
| 24 | 1 | 68 | 1 | 76.5 | 1.74 | 2545.4 |
| 25 | 0 | 71 | 1 | 98.0 | 1.82 | 2716.2 |
| 26 | 1 | 82 | 1 | 91.5 | 1.90 | 3063 |
| 27 | 0 | 83 | 0 | 59.5 | 1.65 | 2314.5 |
| 28 | 1 | 64 | 1 | 65.0 | 1.80 | 2536 |
| 29 | 0 | 73 | 1 | 88.7 | 1.85 | 2799.5 |
| 30 | 1 | 78 | 0 | 50.0 | 1.44 | 1854.4 |
| 31 | 1 | 57 | 0 | 55.3 | 1.53 | 1681.3 |
| 32 | 1 | 77 | 1 | 50.0 | 1.60 | 2560 |
| 33 | 1 | 45 | 0 | 54.5 | 1.51 | 1453.1 |
| 34 | 1 | 78 | 1 | 63.7 | 1.57 | 2542.7 |
| 35 | 1 | 68 | 1 | 73.3 | 1.68 | 2466.8 |
| 36 | 1 | 67 | 0 | 48.0 | 1.55 | 1877.5 |
| 37 | 1 | 48 | 1 | 49.0 | 1.61 | 1935.1 |
| 38 | 1 | 78 | 0 | 54.0 | 1.64 | 2216.4 |
| 39 | 1 | 78 | 1 | 58.0 | 1.70 | 2713 |
| 40 | 1 | 65 | 0 | 56.9 | 1.54 | 1827.4 |
| 41 | 1 | 56 | 1 | 100.0 | 1.95 | 2556.5 |
| 42 | 1 | 54 | 0 | 60.0 | 1.54 | 1651.4 |
| 43 | 1 | 56 | 1 | 67.9 | 1.68 | 2202.8 |
| 44 | 1 | 78 | 1 | 60.0 | 1.58 | 2555.8 |
| 45 | 1 | 78 | 0 | 70.3 | 1.83 | 2560.3 |
| 46 | 1 | 70 | 1 | 62.1 | 1.72 | 2563.2 |
| 47 | 1 | 56 | 1 | 86.9 | 1.83 | 2399.3 |
| 48 | 1 | 64 | 1 | 86.1 | 1.82 | 2562.2 |
| 49 | 1 | 70 | 1 | 85.2 | 1.81 | 2681.1 |
| 50 | 1 | 58 | 1 | 94.0 | 1.85 | 2469.5 |
| 51 | 1 | 66 | 1 | 67.0 | 1.83 | 2619.3 |
| 52 | 1 | 62 | 1 | 63.6 | 1.63 | 2269.3 |
| 53 | 1 | 73 | 1 | 88.8 | 1.72 | 2629.2 |
| 54 | 1 | 78 | 1 | 50.5 | 1.63 | 2621.3 |
| 55 | 1 | 72 | 1 | 105.0 | 1.85 | 2777.5 |
| 56 | 1 | 57 | 1 | 56.9 | 1.73 | 2290.3 |
| 57 | 1 | 78 | 1 | 71.0 | 1.86 | 2922.6 |
| 58 | 1 | 76 | 1 | 69.1 | 1.68 | 2642.8 |

## HFNC Matching Results to Patient Data

Table S8: Matching results to patient data

| **Patient ID** | **RR (bpm)** | **FiO_2_ (%)** | **PaO_2_ (mmHg)** | | | **PaCO_2_ (mmHg)** | | | **∆P_es_ vs. ∆P_pl_ (cmH_2_O)** | | | **Tidal Volume (ml)** | | |
| --- | --- | --- | --- | --- | --- | --- | --- | --- | --- | --- | --- | --- | --- | --- |
|  |  |  | **Data** | **Simulation** | **% Error** | **Data** | **Simulation** | **% Error** | **Data** | **Simulation** | **% Error** | **Data** | **Simulation** | **% Error** |
| 1 | 57 | 45 | 87.30 | 85.78 | 1.7 | 31.00 | 30.79 | 0.7 | 40.00 | 41.47 | 3.7 | 480.7 | 456.6 | 5.0 |
| 2 | 28 | 60 | 67.50 | 67.48 | 0.0 | 34.00 | 33.99 | 0.0 | 32.00 | 31.43 | 1.8 | 616.0 | 614.8 | 0.2 |
| 3 | 45 | 50 | 72.00 | 71.93 | 0.1 | 31.00 | 30.95 | 0.2 | 49.00 | 49.78 | 1.6 | 840.0 | 825.4 | 1.7 |
| 4 | 27 | 40 | 85.00 | 84.45 | 0.6 | 34.00 | 33.01 | 2.9 | 20.00 | 19.97 | 0.1 | 825.0 | 802.3 | 2.7 |
| 5 | 34 | 45 | 76.50 | 76.53 | 0.0 | 39.80 | 39.87 | 0.2 | 42.00 | 41.96 | 0.1 | 751.0 | 751.4 | 0.1 |
| 6 | 50 | 45 | 60.30 | 59.37 | 1.5 | 31.00 | 30.02 | 3.2 | 27.00 | 28.10 | 4.1 | 452.0 | 436.1 | 3.5 |
| 7 | 46 | 55 | 78.57 | 78.16 | 0.5 | 25.00 | 25.34 | 1.3 | 38.00 | 37.82 | 0.5 | 697.0 | 706.3 | 1.3 |
| 8 | 34 | 60 | 60.00 | 59.42 | 1.0 | 36.00 | 36.06 | 0.2 | 38.00 | 37.17 | 2.2 | 882.0 | 860.2 | 2.5 |
| 9 | 42 | 60 | 76.00 | 76.00 | 0.0 | 29.00 | 29.00 | 0.0 | 42.00 | 42.00 | 0.0 | 875.0 | 875.1 | 0.0 |
| 10 | 42 | 85 | 58.40 | 58.76 | 0.6 | 35.00 | 34.45 | 1.6 | 28.00 | 28.77 | 2.7 | 786.0 | 710.1 | 9.7 |
| 11 | 44 | 55 | 71.91 | 69.44 | 3.4 | 33.90 | 33.42 | 1.4 | 22.00 | 23.90 | 8.6 | 727.0 | 576.8 | 20.7 |
| 12 | 20 | 55 | 73.70 | 73.70 | 0.0 | 44.00 | 44.04 | 0.1 | 21.00 | 20.92 | 0.4 | 656.0 | 657.2 | 0.2 |
| 13 | 28 | 50 | 66.25 | 66.59 | 0.5 | 42.00 | 41.94 | 0.1 | 31.00 | 32.00 | 3.2 | 800.0 | 779.2 | 2.6 |
| 14 | 36 | 50 | 61.67 | 61.66 | 0.0 | 38.00 | 38.05 | 0.1 | 35.00 | 34.81 | 0.6 | 578.0 | 578.6 | 0.1 |
| 15 | 26 | 70 | 70.00 | 70.00 | 0.0 | 19.00 | 18.99 | 0.0 | 38.00 | 38.03 | 0.1 | 738.0 | 739.8 | 0.2 |
| 16 | 25 | 40 | 90.00 | 89.94 | 0.1 | 34.00 | 34.00 | 0.0 | 32.00 | 32.04 | 0.1 | 1068.0 | 1069.0 | 0.1 |
| 17 | 26 | 70 | 93.33 | 92.79 | 0.6 | 44.00 | 43.54 | 1.1 | 22.00 | 22.49 | 2.2 | 672.0 | 636.0 | 5.4 |
| 18 | 38 | 60 | 60.00 | 59.94 | 0.1 | 42.00 | 41.98 | 0.1 | 42.00 | 41.99 | 0.0 | 852.0 | 832.2 | 2.3 |
| 19 | 36 | 65 | 60.94 | 60.99 | 0.1 | 37.00 | 37.06 | 0.2 | 39.00 | 38.84 | 0.4 | 653.0 | 652.2 | 0.1 |
| 20 | 45 | 60 | 67.20 | 67.76 | 0.8 | 24.00 | 24.67 | 2.8 | 21.00 | 22.91 | 9.1 | 690.0 | 562.7 | 18.4 |
| 21 | 30 | 60 | 74.40 | 73.60 | 1.1 | 31.00 | 31.15 | 0.5 | 24.00 | 23.92 | 0.4 | 588.0 | 590.1 | 0.4 |
| 22 | 30 | 50 | 97.00 | 96.61 | 0.4 | 40.00 | 39.43 | 1.4 | 29.00 | 32.35 | 11.5 | 1336.0 | 1034.4 | 22.6 |
| 23 | 45 | 85 | 55.25 | 55.14 | 0.2 | 27.00 | 26.84 | 0.6 | 34.00 | 30.83 | 9.3 | 372.0 | 386.8 | 4.0 |
| 24 | 31 | 50 | 85.00 | 85.03 | 0.0 | 42.00 | 41.99 | 0.0 | 38.00 | 37.98 | 0.0 | 834.0 | 834.6 | 0.1 |
| 25 | 25 | 100 | 57.14 | 57.17 | 0.1 | 42.00 | 42.00 | 0.0 | 34.00 | 34.23 | 0.7 | 842.0 | 831.8 | 1.2 |
| 26 | 36 | 70 | 77.00 | 76.37 | 0.8 | 31.00 | 30.40 | 1.9 | 39.00 | 39.49 | 1.3 | 761.0 | 763.1 | 0.3 |
| 27 | 52 | 70 | 70.00 | 70.33 | 0.5 | 31.00 | 31.17 | 0.5 | 42.00 | 38.50 | 8.3 | 622.0 | 521.9 | 16.1 |
| 28 | 25 | 50 | 61.25 | 61.24 | 0.0 | 30.00 | 30.00 | 0.0 | 36.00 | 36.00 | 0.0 | 529.0 | 528.7 | 0.1 |
| 29 | 26 | 55 | 93.50 | 93.47 | 0.0 | 44.00 | 43.98 | 0.1 | 39.00 | 38.96 | 0.1 | 954.0 | 955.3 | 0.1 |
| 30 | 57 | 45 | 87.30 | 86.96 | 0.4 | 40.00 | 40.09 | 0.2 | 37.00 | 35.60 | 3.8 | 526.3 | 540.2 | 2.6 |
| 31 | 45 | 55 | 61.88 | 61.86 | 0.0 | 29.80 | 29.84 | 0.1 | 31.00 | 31.56 | 1.8 | 755.6 | 726.7 | 3.8 |
| 32 | 40 | 60 | 79.20 | 79.07 | 0.2 | 38.00 | 37.82 | 0.5 | 19.00 | 19.44 | 2.3 | 600.0 | 569.2 | 5.1 |
| 33 | 50 | 40 | 79.60 | 80.07 | 0.6 | 39.00 | 38.80 | 0.5 | 22.00 | 23.23 | 5.6 | 600.0 | 553.5 | 7.8 |
| 34 | 34 | 40 | 68.00 | 67.98 | 0.0 | 39.80 | 39.78 | 0.1 | 40.00 | 39.56 | 1.1 | 764.7 | 769.7 | 0.7 |
| 35 | 26 | 40 | 79.20 | 79.25 | 0.1 | 34.00 | 33.87 | 0.4 | 26.00 | 26.03 | 0.1 | 769.2 | 758.2 | 1.4 |
| 36 | 40 | 50 | 71.43 | 71.16 | 0.4 | 34.00 | 34.11 | 0.3 | 36.00 | 35.04 | 2.7 | 650.0 | 644.2 | 0.9 |
| 37 | 36 | 60 | 60.00 | 59.99 | 0.0 | 19.00 | 19.01 | 0.0 | 40.00 | 39.73 | 0.7 | 694.4 | 694.1 | 0.1 |
| 38 | 36 | 70 | 70.00 | 70.05 | 0.1 | 19.00 | 18.99 | 0.0 | 36.00 | 35.77 | 0.6 | 722.2 | 712.2 | 1.4 |
| 39 | 38 | 55 | 69.67 | 69.79 | 0.2 | 42.00 | 41.77 | 0.5 | 44.00 | 43.99 | 0.0 | 657.9 | 658.9 | 0.2 |
| 40 | 42 | 80 | 54.97 | 54.94 | 0.0 | 45.50 | 45.65 | 0.3 | 26.00 | 26.39 | 1.5 | 642.9 | 627.2 | 2.4 |
| 41 | 20 | 45 | 78.75 | 79.15 | 0.5 | 42.00 | 42.07 | 0.2 | 47.00 | 47.30 | 0.6 | 1350.0 | 1337.2 | 1.0 |
| 42 | 28 | 50 | 66.25 | 65.97 | 0.4 | 44.00 | 43.84 | 0.4 | 27.00 | 26.81 | 0.7 | 535.7 | 523.9 | 2.2 |
| 43 | 28 | 55 | 72.88 | 72.64 | 0.3 | 44.00 | 43.94 | 0.1 | 25.00 | 25.18 | 0.7 | 678.6 | 638.0 | 6.0 |
| 44 | 32 | 60 | 74.00 | 74.00 | 0.0 | 40.00 | 40.04 | 0.1 | 35.00 | 34.36 | 1.8 | 650.0 | 654.4 | 0.7 |
| 45 | 28 | 60 | 61.80 | 61.83 | 0.0 | 36.00 | 35.93 | 0.2 | 14.00 | 14.27 | 1.9 | 660.0 | 642.9 | 2.6 |
| 46 | 38 | 50 | 63.34 | 62.89 | 0.7 | 42.00 | 42.09 | 0.2 | 42.00 | 41.73 | 0.6 | 680.0 | 673.8 | 0.9 |
| 47 | 26 | 70 | 70.00 | 69.98 | 0.0 | 19.00 | 18.94 | 0.3 | 38.00 | 37.95 | 0.1 | 816.9 | 813.3 | 0.4 |
| 48 | 32 | 100 | 57.14 | 57.08 | 0.1 | 25.00 | 24.95 | 0.2 | 34.00 | 34.32 | 0.9 | 906.3 | 893.3 | 1.4 |
| 49 | 42 | 65 | 71.50 | 72.05 | 0.8 | 31.00 | 30.92 | 0.3 | 38.00 | 38.37 | 1.0 | 738.1 | 712.4 | 3.5 |
| 50 | 25 | 60 | 58.80 | 59.27 | 0.8 | 29.80 | 29.64 | 0.5 | 32.00 | 31.99 | 0.0 | 1350.0 | 1253.0 | 7.2 |
| 51 | 50 | 45 | 60.00 | 62.05 | 3.4 | 40.00 | 38.95 | 2.6 | 22.00 | 23.98 | 9.0 | 750.0 | 529.7 | 29.4 |
| 52 | 38 | 80 | 60.80 | 60.81 | 0.0 | 40.00 | 39.99 | 0.0 | 42.00 | 42.21 | 0.5 | 700.0 | 692.2 | 1.1 |
| 53 | 32 | 70 | 65.63 | 65.99 | 0.1 | 34.00 | 33.79 | 0.6 | 28.00 | 27.98 | 0.1 | 650.0 | 649.4 | 0.1 |
| 54 | 41 | 55 | 61.60 | 61.60 | 1.0 | 24.00 | 24.19 | 0.8 | 25.00 | 25.66 | 2.6 | 756.1 | 712.1 | 5.8 |
| 55 | 30 | 50 | 62.00 | 61.97 | 0.2 | 31.00 | 30.80 | 0.7 | 24.00 | 25.73 | 7.2 | 1100.0 | 949.1 | 13.7 |
| 56 | 30 | 40 | 70.00 | 69.22 | 0.0 | 30.00 | 29.53 | 1.6 | 25.00 | 26.65 | 6.6 | 1166.7 | 1022.0 | 12.4 |
| 57 | 34 | 90 | 58.50 | 58.71 | 2.2 | 27.00 | 26.97 | 0.1 | 26.00 | 29.42 | 13.1 | 1117.6 | 903.0 | 19.2 |
| 58 | 31 | 60 | 60.00 | 59.81 | 0.7 | 29.00 | 28.61 | 1.4 | 38.00 | 37.98 | 0.1 | 967.7 | 942.6 | 2.6 |

## Simulator Output Table (HFNC Results)

Table S9: Simulator outputs (HFNC results)

| **PID** | **Muscle Pressure (cmH_2_O)** | **PEEP (cmH_2_O)** | **mP_aw_ (cmH_2_O)** | **P/F ratio (mmHg)** | **Resp. Sys. Compliance (ml/cmH_2_O)** | **Lung Compliance (ml/cmH_2_O)** | **Chest Wall Compliance (ml/cmH_2_O)** | **End Exp. Lung Volume (ml)** | **Level of collapse/consolidation (%)** |
| --- | --- | --- | --- | --- | --- | --- | --- | --- | --- |
| 1 | -30.67 | 9.11 | 9.20 | 191 | 14.38 | 16.58 | 108.38 | 3071.8 | 9 |
| 2 | -26.69 | 12.61 | 12.20 | 112 | 27.36 | 34.36 | 134.36 | 3947.8 | 32 |
| 3 | -41.83 | 9.49 | 9.17 | 144 | 20.02 | 24.75 | 104.60 | 3062.0 | 12 |
| 4 | -20.98 | 6.92 | 6.19 | 211 | 55.88 | 85.35 | 161.84 | 3646.3 | 13 |
| 5 | -32.56 | 11.79 | 11.45 | 170 | 22.96 | 28.99 | 110.26 | 3321.3 | 15 |
| 6 | -24.05 | 8.74 | 8.65 | 132 | 19.29 | 23.50 | 107.64 | 3161.9 | 27 |
| 7 | -33.19 | 7.51 | 7.02 | 142 | 20.80 | 26.60 | 95.44 | 2663.7 | 11 |
| 8 | -37.63 | 8.18 | 7.27 | 99 | 32.08 | 40.00 | 162.08 | 3693.9 | 26 |
| 9 | -38.44 | 8.14 | 7.25 | 127 | 27.27 | 35.67 | 115.68 | 3771.2 | 13 |
| 10 | -25.85 | 7.20 | 7.00 | 69 | 28.86 | 37.49 | 125.45 | 3628.9 | 18 |
| 11 | -22.91 | 7.03 | 6.86 | 126 | 29.44 | 37.62 | 135.33 | 3370.3 | 15 |
| 12 | -19.44 | 9.95 | 10.01 | 134 | 41.64 | 57.56 | 150.51 | 3985.1 | 26 |
| 13 | -35.49 | 9.34 | 9.31 | 133 | 30.92 | 39.02 | 148.93 | 3869.4 | 23 |
| 14 | -37.64 | 9.61 | 9.68 | 123 | 20.37 | 25.95 | 94.67 | 2694.5 | 19 |
| 15 | -36.52 | 11.41 | 11.17 | 100 | 27.35 | 35.66 | 117.32 | 3294.4 | 34 |
| 16 | -31.20 | 9.27 | 8.99 | 225 | 41.95 | 58.90 | 145.75 | 4225.3 | 8 |
| 17 | -25.74 | 9.55 | 9.05 | 133 | 39.02 | 53.97 | 140.89 | 3879.2 | 22 |
| 18 | -39.88 | 8.28 | 7.80 | 100 | 26.78 | 33.17 | 139.08 | 3475.5 | 25 |
| 19 | -31.00 | 11.21 | 10.92 | 94 | 22.55 | 29.40 | 96.79 | 3212.8 | 29 |
| 20 | -18.72 | 6.87 | 6.67 | 113 | 27.83 | 36.28 | 119.51 | 3470.3 | 13 |
| 21 | -22.42 | 9.90 | 9.91 | 123 | 26.90 | 36.10 | 105.46 | 3180.4 | 28 |
| 22 | -32.51 | 8.01 | 7.63 | 193 | 37.84 | 50.00 | 155.54 | 4047.0 | 9 |
| 23 | -22.87 | 10.47 | 10.35 | 65 | 18.11 | 21.56 | 113.05 | 2926.8 | 45 |
| 24 | -32.30 | 9.19 | 8.81 | 170 | 30.77 | 39.49 | 139.30 | 3671.0 | 10 |
| 25 | -34.66 | 9.44 | 9.58 | 57 | 32.91 | 42.72 | 143.26 | 3892.4 | 38 |
| 26 | -41.07 | 8.46 | 8.36 | 109 | 25.48 | 31.15 | 140.19 | 4213.2 | 20 |
| 27 | -26.96 | 8.08 | 7.55 | 100 | 19.74 | 25.42 | 88.42 | 3093.5 | 34 |
| 28 | -33.35 | 11.25 | 11.21 | 122 | 22.99 | 27.25 | 147.33 | 3762.3 | 27 |
| 29 | -36.64 | 10.69 | 10.03 | 170 | 33.64 | 43.90 | 143.96 | 3955.3 | 10 |
| 30 | -26.38 | 8.67 | 7.31 | 193 | 21.78 | 29.93 | 80.08 | 2351.8 | 8 |
| 31 | -30.64 | 7.23 | 6.82 | 112 | 26.28 | 35.91 | 98.06 | 2491.1 | 15 |
| 32 | -19.46 | 6.41 | 6.19 | 132 | 33.31 | 45.88 | 121.64 | 3421.0 | 11 |
| 33 | -20.17 | 6.56 | 6.27 | 200 | 27.19 | 36.81 | 104.10 | 2425.7 | 7 |
| 34 | -38.22 | 9.44 | 9.12 | 170 | 25.22 | 32.02 | 118.65 | 3363.2 | 14 |
| 35 | -31.80 | 8.31 | 7.96 | 198 | 39.66 | 56.24 | 134.49 | 3513.6 | 16 |
| 36 | -31.07 | 9.79 | 9.05 | 142 | 24.45 | 33.16 | 93.08 | 2475.6 | 13 |
| 37 | -34.78 | 9.22 | 8.65 | 100 | 26.28 | 32.18 | 143.32 | 3161.3 | 24 |
| 38 | -34.88 | 10.02 | 9.67 | 100 | 25.33 | 35.06 | 91.20 | 2699.1 | 22 |
| 39 | -34.67 | 9.24 | 8.78 | 127 | 22.43 | 27.18 | 128.48 | 3670.3 | 15 |
| 40 | -24.54 | 7.78 | 7.64 | 69 | 26.26 | 36.48 | 93.72 | 2531.8 | 28 |
| 41 | -50.64 | 9.59 | 9.04 | 176 | 41.47 | 55.22 | 166.59 | 4041.2 | 14 |
| 42 | -25.64 | 10.73 | 10.63 | 132 | 27.46 | 37.77 | 100.57 | 2514.3 | 30 |
| 43 | -26.15 | 8.67 | 8.26 | 132 | 37.79 | 51.30 | 143.53 | 3494.0 | 24 |
| 44 | -35.53 | 9.04 | 8.79 | 123 | 25.29 | 32.08 | 119.41 | 3376.7 | 17 |
| 45 | -19.08 | 7.05 | 6.96 | 103 | 43.49 | 75.94 | 101.76 | 3196.6 | 24 |
| 46 | -30.89 | 11.19 | 10.07 | 126 | 25.31 | 31.10 | 136.16 | 3624.1 | 20 |
| 47 | -30.91 | 10.77 | 10.10 | 100 | 32.85 | 41.58 | 156.34 | 3738.8 | 32 |
| 48 | -33.21 | 8.62 | 8.37 | 57 | 30.87 | 38.93 | 148.96 | 3813.7 | 31 |
| 49 | -29.84 | 8.98 | 8.42 | 111 | 25.43 | 30.92 | 143.28 | 3854.9 | 17 |
| 50 | -38.70 | 8.02 | 8.08 | 99 | 44.62 | 62.42 | 156.39 | 3791.8 | 21 |
| 51 | -18.72 | 6.54 | 6.42 | 138 | 26.34 | 32.04 | 148.14 | 3816.7 | 11 |
| 52 | -35.81 | 10.11 | 9.83 | 76 | 22.12 | 26.46 | 134.87 | 3364.9 | 23 |
| 53 | -24.17 | 9.70 | 9.55 | 94 | 27.84 | 35.16 | 133.84 | 3622.1 | 27 |
| 54 | -27.41 | 6.59 | 6.54 | 112 | 30.15 | 39.92 | 123.19 | 3469.3 | 13 |
| 55 | -25.36 | 7.21 | 6.89 | 124 | 41.77 | 58.70 | 144.79 | 4189.5 | 19 |
| 56 | -28.50 | 7.04 | 6.63 | 173 | 43.22 | 61.22 | 147.02 | 3527.5 | 10 |
| 57 | -29.35 | 7.33 | 6.74 | 65 | 34.99 | 46.58 | 140.57 | 3920.6 | 20 |
| 58 | -35.68 | 9.90 | 8.65 | 100 | 34.95 | 48.00 | 128.47 | 3708.3 | 23 |

# **References**

[1] J. G. Hardman, N. M. Bedforth, A. B. Ahmed, R. P. Mahajan, and A. R. Aitkenhead, “A physiology simulator: validation of its respiratory components and its ability to predict the patient’s response to changes in mechanical ventilation.,” *Br J Anaesth*, vol. 81, no. 3, pp. 327–332, 1998.

[2] J. G. Hardman and N. M. Bedforth, “Estimating venous admixture using a physiological simulator,” *Br J Anaesth*, vol. 82, no. 3, pp. 346–349, 1999.

[3] J. G. Hardman and A. R. Aitkenhead, “Estimation of alveolar deadspace fraction using arterial and end-tidal CO2: a factor analysis using a physiological simulation,” *Anaesth Intensive Care*, vol. 27, no. 5, pp. 452–458, 1999.

[4] J. G. Hardman and A. R. Aitkenhead, “Validation of an original mathematical model of CO2 elimination and dead space ventilation,” *Anesth Analg*, vol. 97, no. 6, pp. 1840–1845, 2003.

[5] J. G. Hardman and J. S. Wills, “The development of hypoxaemia during apnoea in children: a computational modelling investigation,” *BJA: British Journal of Anaesthesia*, vol. 97, no. 4, pp. 564–570, 2006.

[6] A. Das, Z. Gao, P. P. Menon, J. G. Hardman, and D. G. Bates, “A systems engineering approach to validation of a pulmonary physiology simulator  for clinical applications.,” *J R Soc Interface*, vol. 8, no. 54, pp. 44–55, Jan. 2011, doi:http://dx.doi.org/10.1098/rsif.2010.0224.

[7] J. G. Hardman and H. M. Al-Otaibi, “Prediction of arterial oxygen tension: validation of a novel formula,” *Am J Respir Crit Care Med*, vol. 182, no. 3, pp. 435–436, 2010.

[8] S. Saffaran, A. Das, J. G. Laffey, J. G. Hardman, N. Yehya, and D. G. Bates, “Utility of Driving Pressure and Mechanical Power to Guide Protective Ventilator  Settings in Two Cohorts of Adult and Pediatric Patients With Acute Respiratory Distress Syndrome: A Computational Investigation.,” *Crit Care Med*, vol. 48, no. 7, pp. 1001–1008, Jul. 2020, doi:http://dx.doi.org/10.1097/CCM.0000000000004372.

[9] L. Weaver *et al.*, “High risk of patient self-inflicted lung injury in COVID-19 with frequently encountered spontaneous breathing patterns: a computational modelling study,” *Ann Intensive Care*, vol. 11, no. 1, pp. 1–8, 2021, doi:http://dx.doi.org/10.1186/s13613-021-00904-7.

[10] L. Weaver *et al.*, “Optimising respiratory support for early COVID-19 pneumonia: a computational modelling study,” *Br J Anaesth*, vol. 128, no. 6, pp. 1052–1058, 2022.

[11] B. Lachmann, “Open up the lung and keep the lung open,” *Intensive Care Med*, vol. 18, no. 6, pp. 319–321, Jun. 1992, doi:http://dx.doi.org/10.1007/BF01694358.

[12] K. G. Hickling, “The pressure-volume curve is greatly modified by recruitment. A mathematical model of ARDS lungs,” *Am J Respir Crit Care Med*, vol. 158, no. 1, pp. 194–202, 1998, doi:http://dx.doi.org/10.1164/AJRCCM.158.1.9708049.

[13] J. H. T. Bates and C. G. Irvin, “Time dependence of recruitment and derecruitment in the lung: a theoretical model,” *J Appl Physiol (1985)*, vol. 93, no. 2, pp. 705–713, 2002, doi:http://dx.doi.org/10.1152/JAPPLPHYSIOL.01274.2001.

[14] B. E. Marshall, W. R. Clarke, A. T. Costarino, L. Chen, F. Miller, and C. Marshall, “The dose-response relationship for hypoxic pulmonary vasoconstriction,” *Respir Physiol*, vol. 96, no. 2–3, pp. 231–247, May 1994, doi:http://dx.doi.org/10.1016/0034-5687(94)90129-5.

[15] J. W. Severinghaus, “Simple, accurate equations for human blood O2 dissociation computations,” *J Appl Physiol Respir Environ Exerc Physiol*, vol. 46, no. 3, pp. 599–602, 1979, doi:http://dx.doi.org/10.1152/JAPPL.1979.46.3.599.

[16] J. W. Severinghaus, “Blood gas calculator,” *J Appl Physiol*, vol. 21, no. 3, pp. 1108–1116, 1966, doi:http://dx.doi.org/10.1152/JAPPL.1966.21.3.1108.

[17] A. R. Douglas, N. L. Jones, and J. W. Reed, “Calculation of whole blood CO2 content,” *https://doi.org/10.1152/jappl.1988.65.1.473*, vol. 65, no. 1, pp. 473–477, 1988, doi:http://dx.doi.org/10.1152/JAPPL.1988.65.1.473.

[18] G. J. McHardy, “The relationship between the differences in pressure and content of carbon dioxide  in arterial and venous blood.,” *Clin Sci*, vol. 32, no. 2, pp. 299–309, Apr. 1967.

[19] G. R. Kelman and J. F. Nunn, “Nomograms for correction of blood Po2, Pco2, pH, and base excess for time and  temperature.,” *J Appl Physiol*, vol. 21, no. 5, pp. 1484–1490, Sep. 1966, doi:http://dx.doi.org/10.1152/jappl.1966.21.5.1484.

[20] O. Siggaard-Andersen, “The van Slyke equation.,” *Scand J Clin Lab Invest Suppl*, vol. 146, pp. 15–20, 1977, doi:http://dx.doi.org/10.3109/00365517709098927.

[21] J. S. Mecklenburgh and W. W. Mapleson, “Ventilatory assistance and respiratory muscle activity. 2: Simulation with an adaptive active (‘aa’ or ‘a-squared’) model lung,” *Br J Anaesth*, vol. 80, no. 4, pp. 434–439, 1998, doi:http://dx.doi.org/10.1093/BJA/80.4.434.

[22] A. Albanese, L. Cheng, M. Ursino, and N. W. Chbat, “An integrated mathematical model of the human cardiopulmonary system: model development,” *American Journal of Physiology-Heart and Circulatory Physiology*, vol. 310, no. 7, pp. H899–H921, Apr. 2016, doi:http://dx.doi.org/10.1152/ajpheart.00230.2014.

[23] E. Agostoni and J. Mead, *Statics of the respiratory system*, vol. 1. American Physiological Soc American Physiological Soc, 1964.

[24] R. Lampe, T. Blumenstein, V. Turova, and A. Alves-Pinto, “Lung vital capacity and oxygen saturation in adults with cerebral palsy,” *Patient Prefer Adherence*, vol. 8, p. 1691, 2014, doi:http://dx.doi.org/10.2147/PPA.S72575.

[25] J. Stocks and P. H. Quanjer, “REFERENCE VALUES FOR RESIDUAL VOLUME, FUNCTIONAL RESIDUAL CAPACITY AND TOTAL LUNG CAPACITY,” *European Respiratory Journal*, pp. 492–506, 1995, doi:http://dx.doi.org/10.1183/09031936.95.08030492.

[26] A. Protti, E. Votta, and L. Gattinoni, “Which is the most important strain in the pathogenesis of ventilator-induced lung injury: dynamic or static?,” *Curr Opin Crit Care*, vol. 20, no. 1, pp. 33–38, Feb. 2014, doi:http://dx.doi.org/10.1097/MCC.0000000000000047.

[27] M. Eikermann and M. F. V. Melo, “Therapeutic range of spontaneous breathing during mechanical ventilation,” *Anesthesiology*, vol. 120, no. 3, p. 536, 2014, doi:http://dx.doi.org/10.1097/ALN.0000000000000126.

[28] M. Cressoni *et al.*, “Mechanical Power and Development of Ventilator-induced Lung Injury,” *Anesthesiology*, vol. 124, no. 5, pp. 1100–1108, May 2016, doi:http://dx.doi.org/10.1097/ALN.0000000000001056.

[29] G. Bellani, A. Grassi, S. Sosio, and G. Foti, “Plateau and driving pressure in the presence of spontaneous breathing,” *Intensive Care Med*, vol. 45, no. 1, pp. 97–98, 2019, doi:http://dx.doi.org/10.1007/S00134-018-5311-9.

[30] M. Okuda *et al.*, “Evaluation by various methods of the physiological mechanism of a high-flow nasal cannula (HFNC) in healthy volunteers,” *BMJ Open Respir Res*, vol. 4, no. 1, pp. 1–10, 2017, doi:http://dx.doi.org/10.1136/bmjresp-2017-000200.

[31] G. A. Plotnikow *et al.*, “Effects of high-flow nasal cannula on end-expiratory lung impedance in semi-seated healthy subjects,” *Respir Care*, vol. 63, no. 8, pp. 1016–1023, 2018, doi:http://dx.doi.org/10.4187/respcare.06031.

[32] A. Corley, L. R. Caruana, A. G. Barnett, O. Tronstad, and J. F. Fraser, “Oxygen delivery through high-flow nasal cannulae increase end-expiratory lung volume and reduce respiratory rate in post-cardiac surgical patients,” *Br J Anaesth*, vol. 107, no. 6, pp. 998–1004, 2011, doi:http://dx.doi.org/10.1093/bja/aer265.

[33] J. Bräunlich, D. Beyer, D. Mai, S. Hammerschmidt, H. J. Seyfarth, and H. Wirtz, “Effects of nasal high flow on ventilation in volunteers, COPD and idiopathic pulmonary fibrosis patients,” *Respiration*, vol. 85, no. 4, pp. 319–325, 2013, doi:http://dx.doi.org/10.1159/000342027.

[34] T. Mündel, S. Feng, S. Tatkov, and H. Schneider, “Mechanisms of nasal high flow on ventilation during wakefulness and sleep,” *J Appl Physiol*, vol. 114, no. 8, pp. 1058–1065, 2013, doi:http://dx.doi.org/10.1152/japplphysiol.01308.2012.

[35] F. Vieira *et al.*, “High-flow nasal cannula compared with continuous positive airway pressure: a bench and physiological study,” *J Appl Physiol*, vol. 132, no. 6, pp. 1580–1590, 2022, doi:http://dx.doi.org/10.1152/japplphysiol.00416.2021.

[36] F. Dalla Corte, I. Ottaviani, G. Montanari, Y. M. Wang, and T. Mauri, “Physiological Effects of High Flow in Adults BT - High Flow Nasal Cannula: Physiological Effects and Clinical Applications,” A. Carlucci and S. M. Maggiore, Eds. Springer International Publishing Springer International Publishing, 2021, pp. 55–65. doi:http://dx.doi.org/10.1007/978-3-030-42454-1_3.

[37] N. Groves and A. Tobin, “High flow nasal oxygen generates positive airway pressure in adult volunteers,” *Australian Critical Care*, vol. 20, no. 4, pp. 126–131, 2007, doi:http://dx.doi.org/10.1016/j.aucc.2007.08.001.

[38] R. Parke, S. McGuinness, and M. Eccleston, “Nasal high-flow therapy delivers low level positive airway pressure,” *Br J Anaesth*, vol. 103, no. 6, pp. 886–890, 2009, doi:http://dx.doi.org/10.1093/bja/aep280.

[39] R. L. Parke, M. L. Eccleston, and S. P. McGuinness, “The effects of flow on airway pressure during nasal high-flow oxygen therapy,” *Respir Care*, vol. 56, no. 8, pp. 1151–1155, 2011, doi:http://dx.doi.org/10.4187/respcare.01106.

[40] J. E. Ritchie, A. B. Williams, C. Gerard, and H. Hockey, “Evaluation of a humidified nasal high-flow oxygen system, using oxygraphy, capnography and measurement of upper airway pressures,” *Anaesth Intensive Care*, vol. 39, no. 6, pp. 1103–1110, 2011, doi:http://dx.doi.org/10.1177/0310057x1103900620.

[41] R. L. Parke and S. P. McGuinness, “Pressures delivered by nasal high flow oxygen during all phases of the respiratory cycle,” *Respir Care*, vol. 58, no. 10, pp. 1621–1624, 2013, doi:http://dx.doi.org/10.4187/respcare.02358.

[42] R. L. Parke, A. Bloch, and S. P. McGuinness, “Effect of very-high-flow nasal therapy on airway pressure and end-expiratory lung impedance in healthy volunteers,” *Respir Care*, vol. 60, no. 10, pp. 1397–1403, 2015, doi:http://dx.doi.org/10.4187/respcare.04028.

[43] S. Komatsu *et al.*, “Increased intrapharyngeal pressure with combined use of high-flow nasal cannula and a surgical face mask: a preliminary study.,” *Fujita medical journal*, vol. 5, no. 4, pp. 104–106, 2019, doi:http://dx.doi.org/10.20407/fmj.2019-004.

[44] E. Garofalo *et al.*, “Evaluation of a new interface combining high-flow nasal cannula and cpap,” *Respir Care*, vol. 64, no. 10, pp. 1231–1239, 2019, doi:http://dx.doi.org/10.4187/respcare.06871.

[45] J. J. Yao, M. M. Peng, G. H. Zou, K. X. He, D. D. Ma, and P. F. Wei, “Effects of flow on carbon dioxide washout and nasal airway pressure in healthy adult volunteers during the constant-flow mode in a non-invasive ventilator,” *Chin Med J (Engl)*, vol. 133, no. 20, pp. 2515–2517, 2020, doi:http://dx.doi.org/10.1097/CM9.0000000000001079.

[46] Y. Miyazaki, S. Inoue, H. Hirose, K. Kooguchi, and J. Kotani, “High-velocity nasal insufflation increases nasopharyngeal pressure with flow-dependent manner compared with high flow nasal cannula in adult volunteers – a single-center prospective observational study,” *Kobe Journal of Medical Sciences*, vol. 67, no. 3, pp. 92–97, 2021.

[47] E. Zhao, Y. Zhou, C. He, and D. Ma, “Factors influencing nasal airway pressure and comfort in high-flow nasal cannula oxygen therapy: a volunteer study.,” *BMC Pulm Med*, vol. 23, no. 1, p. 449, Nov. 2023, doi:http://dx.doi.org/10.1186/s12890-023-02752-6.

[48] J. B. West, *Respiratory physiology: the essentials*. Lippincott Williams & Wilkins Lippincott Williams & Wilkins, 2012.

[49] A. B. Lumb, “Distribution of pulmonary ventilation and perfusion,” *Nunn’s applied respiratory physiology*, pp. 119–144, 2010.

[50] W. Möller *et al.*, “Nasal high flow clears anatomical dead space in upper airway models,” *J Appl Physiol*, vol. 118, no. 12, pp. 1525–1532, 2015, doi:http://dx.doi.org/10.1152/japplphysiol.00934.2014.

[51] W. Möller *et al.*, “Nasal high flow reduces dead space,” *J Appl Physiol*, vol. 122, no. 1, pp. 191–197, 2017, doi:http://dx.doi.org/10.1152/japplphysiol.00584.2016.

[52] M. I. Pinkham *et al.*, “Effect of respiratory rate and size of cannula on pressure and dead-space clearance during nasal high flow in patients with COPD and acute respiratory failure,” *J Appl Physiol (1985)*, vol. 132, no. 2, pp. 553–563, 2022, doi:http://dx.doi.org/10.1152/japplphysiol.00769.2021.

[53] M. Laviola, A. Das, M. Chikhani, D. G. Bates, and J. G. Hardman, “Computer simulation clarifies mechanisms of carbon dioxide clearance during apnoea,” *Br J Anaesth*, vol. 122, no. 3, pp. 395–401, 2019, doi:http://dx.doi.org/https://doi.org/10.1016/j.bja.2018.11.012.

[54] R. Tonelli *et al.*, “Early Inspiratory Effort Assessment by Esophageal Manometry Predicts Noninvasive Ventilation Outcome in De Novo Respiratory Failure: A Pilot Study,” *Am J Respir Crit Care Med*, vol. 202, no. 4, pp. 558–567, 2020, doi:http://dx.doi.org/10.1164/rccm.201912-2512OC.

[55] R. Tonelli *et al.*, “Inspiratory Effort and Lung Mechanics in Spontaneously Breathing Patients with Acute Respiratory Failure due to COVID-19: A Matched Control Study.,” *American journal of respiratory and critical care medicine*, vol. 204, no. 6. United States, pp. 725–728, Sep. 2021. doi:http://dx.doi.org/10.1164/rccm.202104-1029LE.
